# OTOP2 proton channel couples luminal pH sensing to intestinal immune homeostasis

**DOI:** 10.1016/j.jbc.2026.113105

**Published:** 2026-05-07

**Authors:** Weihui Yan, Ying Wang, Hongxia Zhao, Shicheng Peng, Ying Lu, Bo Wu, Yongtao Xiao

**Affiliations:** 1 Division of Pediatric Gastroenterology and Nutrition, Xinhua Hospital, School of Medicine, Shanghai Jiao Tong University, Shanghai, China; 2 Shanghai Key Laboratory of Pediatric Gastroenterology and Nutrition, Shanghai, China; 3 Shanghai Institute of Pediatric Research, Shanghai, China; 4 Department of Pediatric Surgery, Hangzhou Children’s Hospital, Hangzhou, Zhejiang, China

**Keywords:** OTOP2, pH sensing, Paneth cells, goblet cells, intestinal barrier

## Abstract

Extracellular acidification is a hallmark of the inflamed intestinal tract in individuals with inflammatory bowel disease (IBD). The proton channel Otopetrin-2 (*OTOP2*), which is enriched in intestinal tissues, becomes dysregulated during inflammation. However, the specific role of *OTOP2* in IBD pathogenesis remains unclear. In this study, we demonstrate a significant reduction of *OTOP2* mRNA and protein in the inflamed mucosa of pediatric patients with IBD, which inversely correlates with disease progression. *Otop2* knockout (*Otop2*^*−/−*^) mice exhibit growth retardation and heightened susceptibility to intestinal inflammation, which is linked to intestinal pH dysregulation, altered gut microbiota composition, and compromised tight junction integrity. Accordingly, *Otop2*^*−/−*^ mice exhibit increased susceptibility to dextran sulfate sodium (DSS)-induced colitis. Mechanistically, *Otop2* deficiency reduces Paneth cell numbers and diminishes antimicrobial factor expression, likely due to impaired autophagy-lysosomal processes within these cells. Similarly, *Otop2* deficiency impairs phagocytic function in bone marrow-derived macrophages (BMDMs). Together, these findings establish OTOP2 as a critical, pH-sensitive regulator of intestinal homeostasis and highlight its potential as a therapeutic target in IBD.

Inflammatory bowel disease (IBD), encompassing Crohn’s disease and ulcerative colitis, is a chronic, relapsing-remitting inflammatory disorder of the gastrointestinal tract (1). Once considered a disease of the Western world, IBD is now recognized as a global health challenge with a rising incidence worldwide (2). The epidemiological shift reveals a distinct geographical pattern: while incidence rates in developed nations have plateaued at 25 to 40 cases per 100,000 person-years, newly industrialized and developing regions are experiencing a rapid increase from previously low baselines (1–10 per 100,000 person-years) (3). This expanding burden underscores the urgent need to identify novel molecular targets for therapeutic intervention. The pathogenesis of IBD is complex and heterogeneous, arising in genetically susceptible individuals from a dysregulated immune response to the gut microbiome. Disease manifestation is driven by the convergence of genetic, immune, environmental, and microbial factor (4). Despite the expanding therapeutic options for IBD, which encompass anti-TNF agents, anti-α4β7 integrin, anti-IL-12/IL-23 agents, Janus kinase inhibitors, and sphingosine-1-phosphate receptor modulators, treatment response remains suboptimal (5). The current gap in the therapeutic landscape is lack of strategies designed to directly promote epithelial repair, restore barrier integrity, and correct microbiota dysbiosis. An approach that integrates these fundamental aspects of IBD pathophysiology could offer a transformative, complementary strategy to existing immunomodulatory regimens.

The gastrointestinal epithelium is a single layer of cells that constitutes a critical, semi-permeable barrier between the host and the luminal environment, with essential roles in host defense, nutrient absorption, and immune coordination. In inflammatory bowel disease (IBD), intestinal epithelial cell (IEC) homeostasis is profoundly disrupted (6, 7). This dynamic barrier is maintained by specialized IEC lineages, including absorptive enterocytes/colonocytes and secretory cells such as goblet cells (GCs), Paneth cells (PCs), and enteroendocrine cells (8). A hallmark of epithelial dysfunction in IBD, particularly in ulcerative colitis, is a notable depletion of GCs (9). GCs are indispensable for barrier integrity through their dual functions of secreting mucins and facilitating antigen sampling and presentation to underlying dendritic cells (DCs) (10). The secreted mucins and antimicrobial peptides (AMPs) form a formidable physical and chemical barrier. In the colon, this results in a stratified mucus layer, with the inner layer remaining largely sterile (11). In the small intestine, sterility is primarily maintained by AMPs secreted by PCs (12). PCs are specialized intestinal epithelial cells (IECs) located at the bases of small-intestinal crypts and serve as vital custodians of intestinal homeostasis (12, 13, 14). Several mechanisms link epithelial barrier failure to intestinal inflammation, including defects in autophagy (8) and inflammasome dysfunction (15). Notably, compromised autophagy within PCs disrupts the expression and secretion of antimicrobial factors and skews intestinal immune responses (16, 17, 18). In addition to the epithelium, mucosal macrophages play a pivotal role in maintaining homeostasis by orchestrating inflammation resolution and facilitating tissue repair (19). A key macrophage function is phagocytosis, wherein pathogens are internalized and degraded within acidified intracellular compartments (20). Impairments in this process can thus critically compromise innate immune defense in the gut.

The Otopetrin (*OTOP*) family comprises evolutionarily conserved, proton-selective ion channels, with three known members in vertebrates: *OTOP1*, *OTOP2*, and *OTOP3*. While structurally related, these channels exhibit distinct tissue expression profiles and functions (21). *OTOP1* is predominantly localized within taste and vestibular cells, brown adipose tissue, the mammary gland, and cardiac tissues (21, 22), and has been established as the primary receptor for sour taste perception (23, 24). *OTOP3* is found in the epidermis, stomach lining, and retina (25). In contrast, *OTOP2* is mainly located at intestinal tract and co-expressing with the chloride channel bestrophin 4 (BEST4) in mature colonocytes (25). However, the functional roles of *OTOP2* remain insufficiently explored and largely enigmatic at this stage. It is well-established that a low pH within the gastrointestinal tract correlates with the hallmark features of active IBD (26). Elevated local proton concentrations, driven by factors such as heightened glycolytic metabolism and short-chain fatty acid (SCFA) synthesis, contribute to luminal and mucosal acidification in patients (8, 27, 28). Our preliminary investigations revealed that *OTOP2* is progressively downregulated in the inflamed intestinal mucosa of pediatric patients with IBD. This inverse correlation suggests that *OTOP2*, as a proton channel, may be intricately linked to IBD pathogenesis, potentially acting as a pH-sensitive regulator of intestinal homeostasis. In the present study, we sought to elucidate the specific roles and mechanisms of *OTOP2* in maintaining intestinal homeostasis. We first confirmed that *OTOP2* is enriched in gut epithelial cells. We then generated *Otop2* knockout (*Otop2*^*−/−*^) mice to systematically examine the impact of its deficiency on intestinal inflammation, barrier integrity, and gut microbiota. Given the established link between proton handling and lysosomal function, we further investigated whether *OTOP2* regulates key cellular processes such as autophagy in Paneth cell and macrophage phagocytosis. Our findings suggests that OTOP2 plays a pivotal role in sustaining intestinal homeostasis, likely through its capacity for pH-sensing and lysosomal function, which is seen as attractive therapeutic target in IBD.

## Results

### OTOP2 is diminished in pediatric inflammatory bowel disease

We firstly to detect the expression profile of *Otop2* gene using quantitative reverse-transcription PCR (qRT-PCR) in organs of the mice and found *Otop2* mRNA was predominantly expressed in bone marrow, spleen, and mucosa of the gastrointestinal tract (Fig. S1*A*). Immunofluorescence (IF) staining confirmed that OTOP2 protein was present within the mucosal layers of mice proximal (*pro*), middle (*mid*), and distal (*dis*) small intestine segments and colons (Fig. S1, *B* and *C*). Notably, levels of *Otop2* mRNA in murine intestines increased beginning on postnatal day 0 (P0; Fig. S1*D*). In a similar vein, the expression pattern of the Paneth cell markers *Lyz1* and *Mptx2* mirrored this trend over time as indicated (Fig. S1*D*). In alignment with these observations, Immunofluorescence (IF) analysis also indicated that OTOP2 protein was co-localized with the Paneth cell marker lysozyme at crypts’ basements of both mice (Fig. S1*E*). Single-cell mRNA sequencing in human small intestines and colons unveiled that *OTOP2* mRNA is not only enriched in Paneth cells but also in intestinal macrophages (Fig. S2) (29, 30).

**Figure 1:**
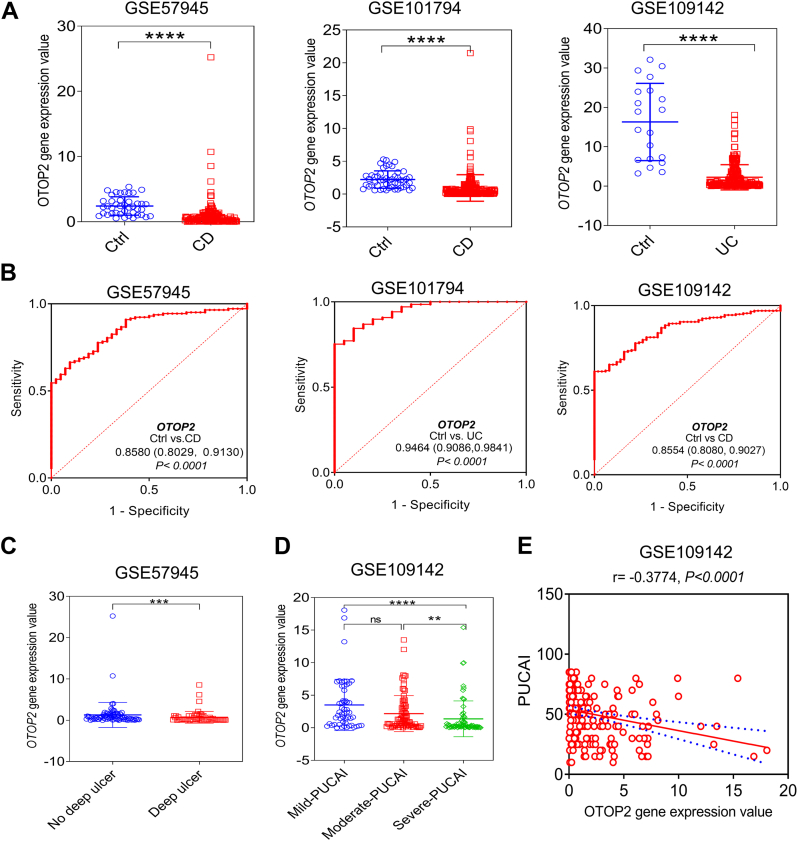
***OTOP2* mRNA expression levels are linked to disease severity of IBD children.***A*, data for *OTOP2* mRNA expression extracted from the GEO database, comparing intestinal tissues from patients with Crohn’s disease (CD) or ulcerative colitis (UC) and non-IBD control subjects. GSE57945, CD, n = 143, control, n = 42; GSE101794, CD, n = 198, control, n = 50; GSE109142, UC, n = 206, control, n = 20. *B*, the AUC for *OTOP2* in differentiating UC or CD patients and non-IBD patients. The AUC value is shown with 95% CI. Data were extracted from the datasets GSE57945, GSE101794, and GSE109142. *C*, the mRNA expression levels of *OTOP2* between CD children with or without deep ulcer. *D*, the mRNA expression levels of *OTOP2* in UC children with different disease severity. *E*, the mRNA expression levels of *OTOP2* was correlated with PUCAI in UC children. Data were extracted from the dataset GSE109142. Mild-PUCAI, n = 54; Moderate-PUCAI, n = 83; Severe-PUCAI, n = 69. Data presented in (*A*, *C*, and *D*) was expressed as the mean ± standard deviation (SD). Non-parametric Mann–Whitney *U* test was for (*A* and *C*). The Kruskal–Wallis test followed by Dunn's multiple comparisons test for (*D*). Statistical significance: ∗∗*p* < 0.01; ∗∗∗*p* < 0.001; ∗∗∗∗*p* < 0.0001; ns, not significant. Abbreviation: Ctrl, control; PUCAI, pediatric ulcerative colitis activity index; AUC, area under the curve; CD, Crohn’s disease, IBD, inflammatory bowel disease; UC, ulcerative colitis.

**Figure 2:**
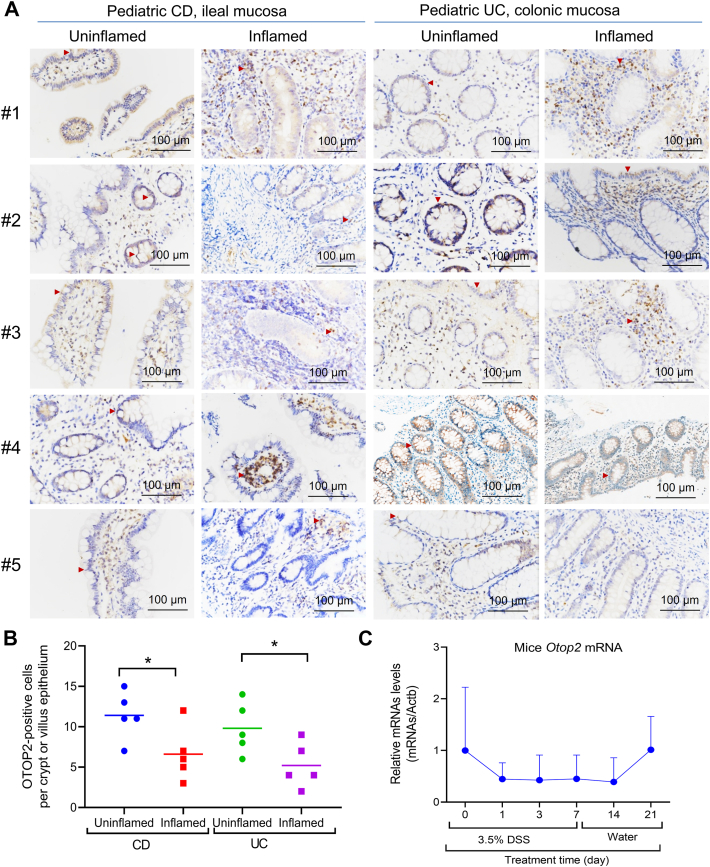
**OTOP2 protein decreasingly expressed in inflamed tissues of pediatric IBD.***A*, representative Immunohistochemistry (IHC) images of OTOP2 in intestinal tissues of pediatric patients with CD and UC. Quantification of OTOP2 IHC staining (each group, n = 5). *B*, unpaired two-tailed Student’s *t* test with or without Welch’s correction analysis for (*A*). *C*, qRT-PCR analysis of *Otop2* mRNA expression in colons of mice (n = 5–14) subjected to DSS-induced colitis and recovery phases. Data presented in (*B* and *C*) was expressed as the mean ± standard deviation (SD). Statistical significance: ∗*p* < 0.05; Abbreviation: CD, Crohn’s disease; CD, Crohn’s disease; UC, ulcerative colitis; OTOP2, otopetrin 2; qRT-PCR, quantitative real-time PCR, IHC, immunohistochemistry.

**Figure 3:**
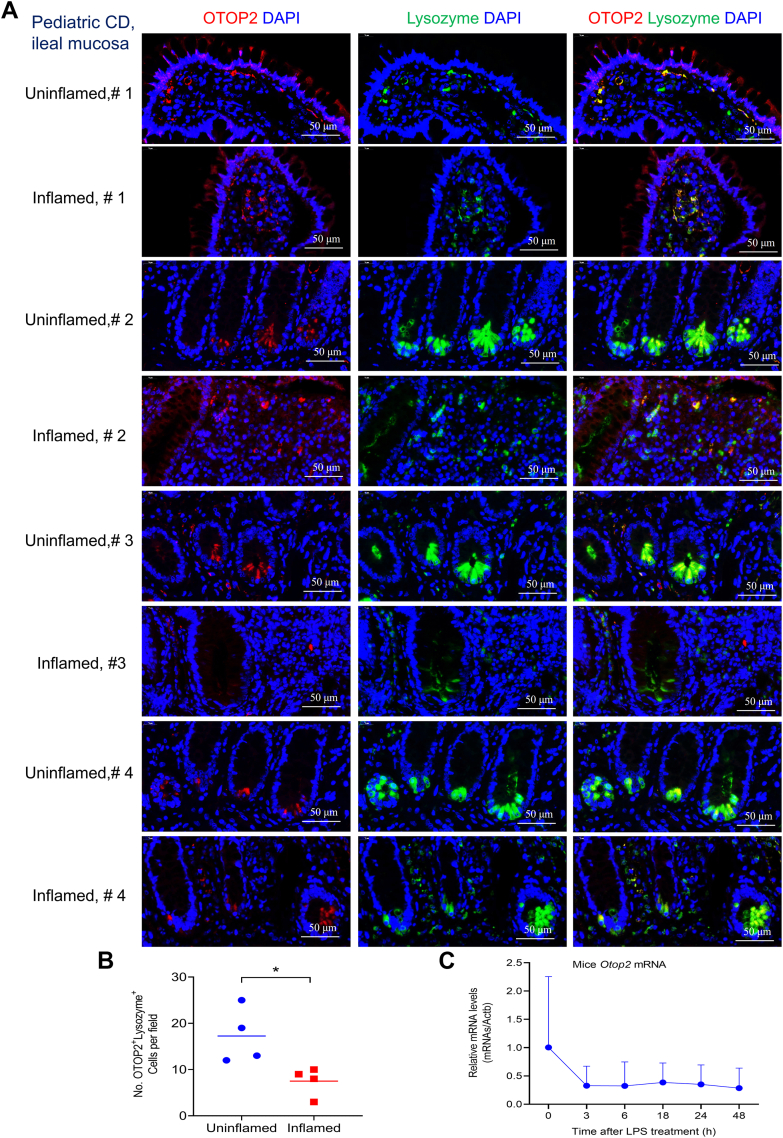
**OTOP2 reduced in Paneth cells during the intestinal inflammation.***A*, representative images of immunofluorescence (IF) staining for OTOP2 and Lysozyme in ileal mucosa from pediatric patients with Crohn's disease (CD, n = 4). *B*, quantification of OTOP2 and Lysozyme positive cells in panel (*A*). OTOP2 (*red*), Lysosome (*green*) and 4′,6-diamidino-2-phenylindole (DAPI, *blue*). *C*, quantitative real-time PCR (qRT-PCR) of *Otop2* mRNA levels in small intestines of mice during lipopolysaccharide (LPS) stimulation from 0 to 48 h (each time point, mice, n = 8–12). Unpaired two-tailed Student’s *t* test with or without Welch’s correction analysis for (*B*). Data presented in (*B* and *C*) was expressed as the mean ± standard deviation (SD). Statistical significance: ∗*p* < 0.05.

Analysis of publicly available datasets revealed a significant downregulation of *OTOP2* mRNA in ileal tissues from pediatric patients with Crohn's disease (CD) compared to healthy controls (Fig. 1*A*) (31, 32). The diagnostic potential of *OTOP2* for distinguishing CD from controls was high, with area under the curve (AUC) values ranging from 0.8029 to 0.9130 (Fig. 1*B*) (31, 32). Notably, *OTOP2* expression was further reduced in CD patients who progressed to complicated diseases with deep ulcers compared to those who did not (Fig. 1*C*) (31). A similar pattern was observed in pediatric ulcerative colitis (UC), where OTOP2 mRNA levels were markedly lower in inflamed tissues than in controls (Fig. 1*A*) (33). Receiver operating characteristic (ROC) analysis confirmed its strong diagnostic utility for UC, with AUCs between 0.9086 and 0.9841 (Fig. 1*B*) (33). Furthermore, in rectal tissues, OTOP2 expression exhibited an inverse correlation with disease severity, progressively declining as Pediatric Ulcerative Colitis Activity Index (PUCAI) scores increased (Fig. 1, *D* and *E*) (33). Subsequently, we evaluated the expression profile of *OTOP2* protein within intestinal tissues derived from patients afflicted with CD and UC through immunohistochemistry (IHC) staining. Consistent with the transcriptomic data, the number of OTOP2-positive cells in the villi and crypts was significantly reduced in inflamed mucosa from both CD and UC patients compared to uninflamed controls (Fig. 2, *A* and *B*). In a dextran sulfate sodium (DSS)-induced mouse model of colitis and recovery, colonic *Otop2* expression was sharply downregulated during the acute inflammatory phase but rebounded during the recovery period (Fig. 2*C*). Immunofluorescence (IF) staining further illustrated a reduced co-expression frequency between *OTOP2* protein and lysozyme cells situated in inflamed ileum samples obtained from pediatric CD cases compared to their uninflamed counterparts (Fig. 3, *A* and *B*). Furthermore, in lipopolysaccharide (LPS)-induced murine model of intestinal inflammation, the mRNA levels of *Otop2* plummeted to their nadir 3 hours post-LPS administration and remained significantly suppressed for 48 h within the small intestinal mucosa (Fig. 3*C*). In human colonic epithelium, *OTOP2* has been found to co-localize with chloride channel bestrophin 4 (*BEST4*) (25). We here showed that BEST4 protein co-expressed with OTOP2 within the pediatric colonic mucosa and diminished in inflamed mucosa of pediatric IBD (Fig. S3, *A*–*C*). We also analyzed the *BEST4* mRNA expression in public datasets (31, 32, 33). As shown in Fig. S4, the BEST4 has similar expressed patterns as that observed *OTOP2* in IBD patients (Fig. S4).

### Otop2 deficiency predisposes mice to intestinal inflammation

**Figure 4:**
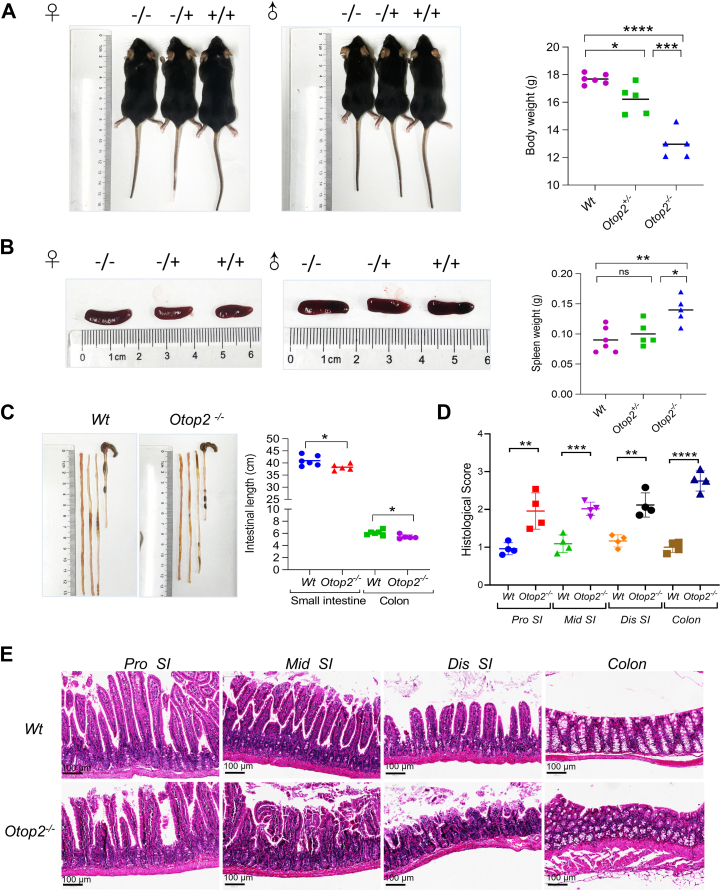
**Phenotyping of *Otop2* knockout (*Otop2*^*−/−*^) mice.***A*, representative images of *Otop2*^*−/−*^; *Otop2*^*−/*+^ and, *Wt* mice. Quantification of body weight of *Otop2*^*−/−*^; *Otop2*^*−/*+^ and, *Wt* mice (each group, n = 5–6). *B*, representative images of spleens from *Otop2*^*−/−*^; *Otop2*^*−/*+^ and, *Wt* mice. Quantification of spleens’ weight of *Otop2*^*−/−*^, *Otop2*^*−/*+^ and, *Wt* mice (each group, n = 5–6). *C*, representative images of small intestines and colons from *Otop2*^*−/−*^ (n = 5) mice and their *Wt* (n = 6) littermates. Quantification of the length of intestines from *Otop2*^*−/−*^ and *Wt* mice. (*D*) Quantification of panel (*E*). *E*, representative images of histology for proximal (*pro*), middle (*mid*), distal (*dis*) small bowel and colon of *Otop2*^*−/−*^ and *Wt* mice (each group, n = 4). Data presented in (*A*, *B*, *C* and *D*) was expressed as the mean ± standard deviation (SD). Ordinary One-way ANOVA followed by Tukey's multiple comparisons test for (*A*, *B*). Unpaired two-tailed Student’s *t* test analysis for (*C*, *D*). Statistical significance: ns, not significant, ∗*p* < 0.05, ∗∗*p* < 0.01, ∗∗∗*p* < 0.001, ∗∗∗∗*p* < 0.0001; −/−, *Otop2*^*−/−*^, −/+, *Otop2*^*−/*+^; +/+, *Wt* Abbreviation: *Wt*, *Wild type*; *Otop2*^*−/−*^, *Otop2* knockout Homozygote*; Otop2*^*−/+*^, *Otop2* knockout *Heterozygote*.

**Figure 5:**
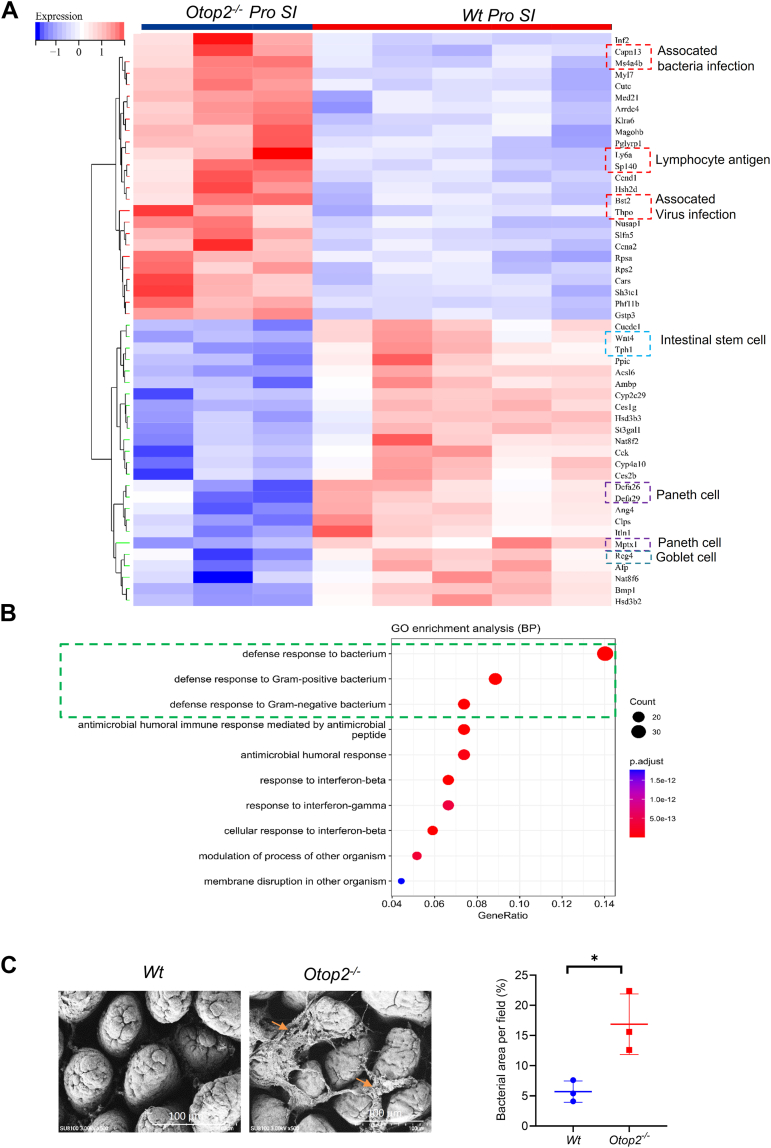
**The differentially expressed genes in the proximal (*pro*) small intestinal mucosa of *Otop2*^*−/−*^ mice and *Wt* mice.***A*, heatmap of differentially expressed genes in the proximal (pro) small intestines of *Otop2*^*−/−*^ mice (n = 3) and *Wt* mice (n = 5). *B*, gene Ontology (GO) enrichment analysis results of differentially expressed genes in the small intestines of *Otop2*^*−/−*^ mice and *Wt* mice. *C*, representative images of scanning electron microscopy (SEM) analysis for the small intestinal mucosa of both *Otop2*^*−/−*^ and *Wt* mice. Quantification of percent of bacterial area per field (mice, each group, n = 3). Data presented was expressed as the mean ± standard deviation (SD). Unpaired two-tailed Student’s *t* test with or without Welch’s correction analysis for (*C*). Statistical significance: ∗*p* < 0.05. Abbreviation: *SI*, small intestinal mucosa; *Wt,Wild type*; SEM, scanning electron microscopy.

To investigate the exact roles of *Otop2* in intestinal homeostasis, we initially generated a cohort of mice deficient in the *Otop2* gene (*Otop2*^*−/−*^, Fig. S5). As illustrated in Figure 4, the *Otop2*^*−/−*^ mice exhibited significant systemic growth retardation, characterized by reduced stature and diminished body weight when compared to their *Wild type* (*Wt*) or heterozygous (*Otop2*^*−/+*^) counterparts (Fig. 4*A*). This suggests a potential global metabolic or nutrient absorption defect. These mutant mice displayed splenomegaly (increased spleen mass) as well as brain enlargement relative to relative to that of *Wt* mice (Figs. 4*B* and S6*A*), indicative of possible systemic immune activation or developmental perturbations. Consistently, *Otop2*^*−/−*^ mice exhibited increased inflammatory infiltrates in multiple organs, including the brain, liver, pancreas, stomach, kidney, and spleen, relative to *Wt* or *Otop2*^*−/+*^ mice (Fig. S6*B*). Upon assessing the intestines of these subjects, it became evident that *Otop2*^*−/−*^ mice possessed significantly shortened small intestines and colons than their *Wt* counterparts (Fig. 4*C*), a hallmark of impaired intestinal development or adaptation. Histological scoring further indicated that abnormalities were present within villi and crypts; notably, heightened immune infiltration was observed within the intestines of *Otop2*^*−/−*^ mice compared to those from *Wt* controls (Fig. 4, *D* and *E*). The RNA sequencing to elucidate the genes affected within the small intestinal and colonic mucosa. In proximal (*pro*) small intestines of *Otop2*^*−/−*^ mice, our findings revealed a downregulation of genes associated with intestinal stem cells, enteroendocrine cells, Paneth cells, and goblet cells. Conversely, genes linked to bacterial or viral infections exhibited an upregulation in the proximal small intestine when compared to *Wt* mice (Fig. 5*A*). Our comprehensive analysis indicated that within the small intestinal mucosa, the most significantly altered genes were notably enriched in processes related to defense responses against bacteria, antimicrobial activities, and secretory granule formation (Fig. 5*B*). Indeed, the scanning electron microscopy (SEM) analysis revealed a marked increase in the number of invading bacteria that adhered to and aggregated upon the epithelial surface within the small intestines of *Otop2*^*−/−*^ mice (Fig. 5, *C* and *D*). In distal (dis) small intestines of *Otop2*^*−/−*^ mice, the RNA sequencing showed that most altered genes were enriched in pathways of inflammatory response, fatty acid metabolic process, and cellular response to xenobiotic stimulus (Fig. S7). In contrast, when examining the colon mucosa relative to *Wt* mice, we found that *Otop2*^*−/−*^ mice displayed a pronounced enrichment of altered genes involved in ion regulation or cation transmembrane transport as well as cellular contraction and extracellular matrix organization (Fig. S8).

### Otop2 deficiency disrupts luminal pH and gut microbiota composition

**Figure 6:**
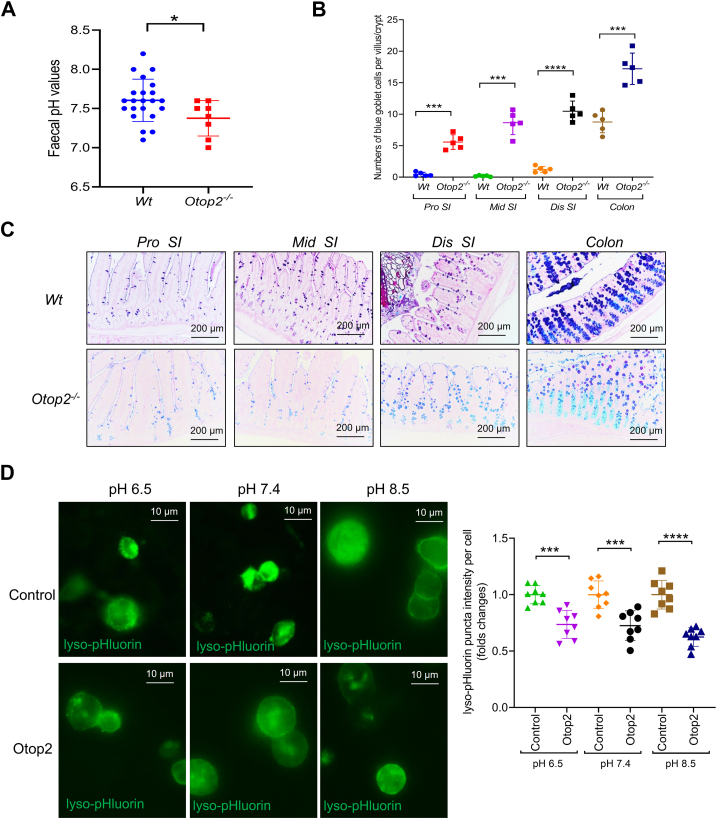
***Otop2* knockout impaired pH sensing in mice intestinal tracts.***A*, Measuring the fecal pH values of *Otop2*^*−/−*^ mice (n = 8) and *Wt* mice (n = 22). *B*, quantification of goblet cells number (stained blue) in both *Otop2*^*−/−*^ mice (n = 5) and *Wt* mice (n = 5). *C*, representative images of Alcian blue/periodic acid Schiff base (AB-PAS) staining for the proximal (*pr*o), middle (*mid*), and distal (*dis*) small bowel from both *Otop2*^*−/−*^ mice and *Wt* mice. *D*, HEK-293 cells expressing Otop2 and a genetically encoded pH sensor lyso-pHluorin were stimulated with different pH (6.5–8.5), and the lyso-pHluorin puncta were imaged. Quantification of the average intensities of lyso-pHluorin puncta in presence of Otop2 or not (each group, n = 8). lyso-pHluorin (*green*); Data presented in (*A*, *B* and *D*) was expressed as the mean ± standard deviation (SD). Unpaired two-tailed Student’s *t* test with or without Welch’s correction analysis for (*A*, *B* and *D*). Statistical significance: ∗*p* < 0.05, ∗∗∗*p* < 0.001, ∗∗∗∗*p* < 0.0001.

**Figure 7:**
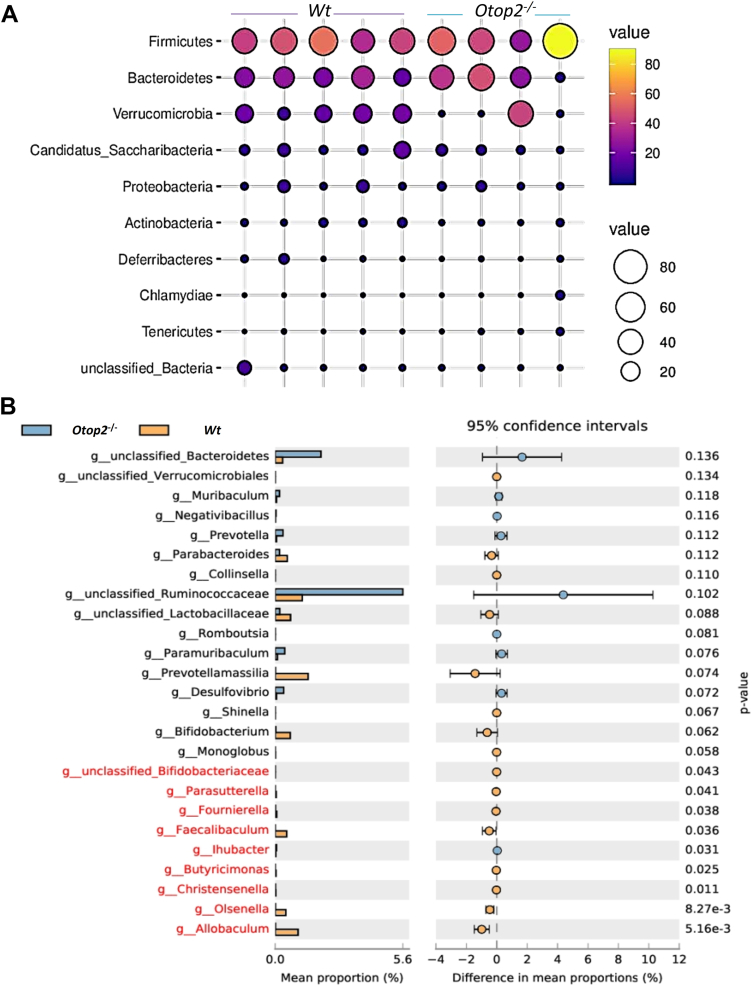
***Otop2* deficiency altered the bacteria composition in feces.***A*, the relative abundance of the top bacteria (phylum) in the feces of *Otop2*^*−/−*^ mice (n = 4) and *Wt* mice (n = 5). *B*, the relative abundance of the top bacteria (genus) in the feces of *Otop2*^*−/−*^ mice (n = 4) and *Wt* mice (n = 5). Unpaired two-tailed Student’s *t* test with or without Welch’s correction analysis for (*B*); *Red* marked, *p* values < 0.05.

As indicated Figure 6*A*, the fecal pH values were significantly reduced in *Otop2*^*−/−*^ mice relative to their *Wt* counterparts (Fig. 6*A*). Consistent with these observations, the alcian blue-periodic acid-Schiff (AB-PAS) staining demonstrated a substantial rise in blue goblet cells within both the small intestines and colons of *Otop2*^*−/−*^ mice when compared to those from *Wt* mice-indicative of mucus transitioning towards acidity following *Otop2* knockout (Fig. 6, *B* and *C*). Lyso-pHluorin serves as a pH sensor for endolysosomes, whose fluorescence is quenched under acidic conditions but activated upon neutralization or deacidification within endolysosomal compartments (34). We sought to determine whether lyso-pHluorin could function effectively as a pH sensor for proton channel *Otop2* (21). As illustrated in Figure 6, *D* and *E*, overexpression of *Otop2* resulted in decreased formation of lyso-pHluorin puncta within HEK-293 cells subjected to varying pH stimuli -thereby confirming that *Otop2* facilitates proton transport into cellular structures or vesicles (Fig. 6, *D* and *E*). Subsequently, we employed 16S rRNA sequencing to investigate the luminal bacterial composition in both *Otop2*^*−/−*^ and *Wt* mice (Fig. S9). In comparison with their *Wt* littermates, *Otop2*^*−/−*^ mice showed fewer operational taxonomic units (OTUs) in the phylum *Verrucomicrobia*, *Candidatus* and *Actinobacteria* (Figs. 7*A* and S9). Notably, there was a reduction in OTUs in the genera Bifidobacteriaceae, *Parasutterella*, *Foumierella*, *Faecalibaculum*, *Ihubacter*, *Butyricimonas*, *Christensenella*, *Olsenella*, and *Allobaculum* decreased significantly, but *Bacteroidetes*, *Prevotella*, Ruminococcaceae in increased in *Otop2*^*−/−*^ mice (Figs. 7*B* and S9). Combined analysis of 16S rRNA sequencing and transcriptomics showed altered abundance of these bacteria were correlated with the expression levels of genes involved in bacterial defense, inflammatory response, fatty acid metabolic process, and cellular response to xenobiotic stimulus (Figs. S10–S12).

### Loss of Otop2 impaired intestinal barrier integrity and proliferation

**Figure 8:**
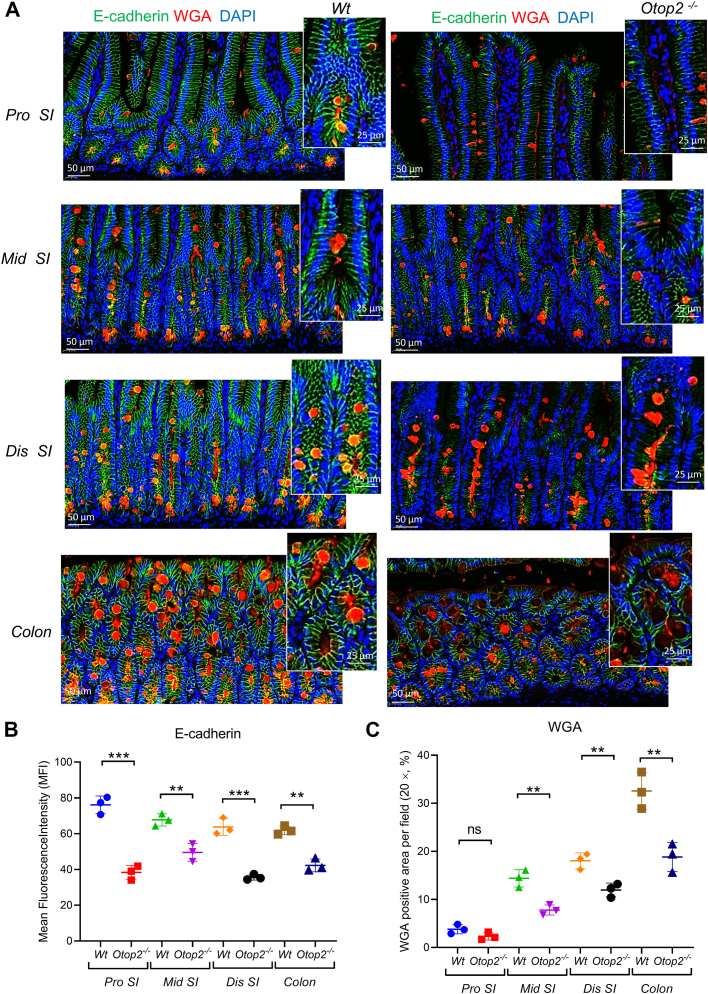
***Otop2* deficiency reduced E-cadherin and goblet mucus.***A*, representative images of immunofluorescence (IF) staining of E-cadherin (*green*) and wheat germ agglutinin (WGA, *red*) for the proximal (*pro*), middle (*mid*), distal (*dis*) small bowel and colon from both *Otop2*^*−/−*^ mice (n = 3) and *Wt* mice (n = 3). *B, C*, quantification of E-cadherin and WGA in panel (*A*). Data presented in (*B* and *C*) was expressed as the mean ± standard deviation (SD). Unpaired two-tailed Student’s *t* test with or without Welch’s correction analysis for (*B* and *C*). Statistical significance: ns, not significant, ∗∗*p* < 0.01, ∗∗∗*p* < 0.001.

**Figure 9:**
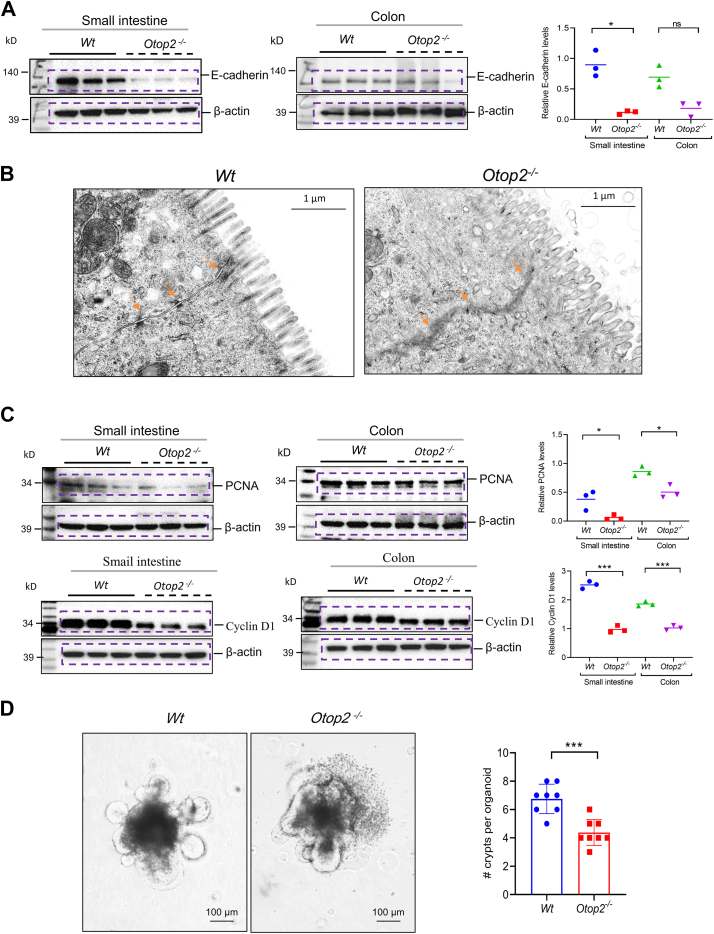
***Otop2* deficiency impaired the tight junction and crypt growth.***A*, Western blotting (WB) analysis for E-cadherin in mucosa of small bowel and colon from *Otop2*^*−/−*^ mice and *Wt* mice (each group, n = 3). *B*, transmission electron microscopy (TEM) analysis tight junctions in intestines of *Otop2*^*−/−*^ mice and *Wt* mice. *C*, Representative images of WB analysis for proliferating cell nuclear antigen (PCNA) and Cyclin D1 protein in both small intestinal and colonic mucosa of *Otop2*^*−/−*^ mice and *Wt* mice (each group, n = 3). Quantification of Pcna expression. *D*, representative images of intestinal crypt budding in organoids from both *Otop2*^*−/−*^ mice (n = 3) and *Wt* mice (n = 3) at fifth day of culture. Qualification of crypt budding in organoids. Each group, n = 3; Data presented in (*A*, *C* and *D*) was expressed as the mean ± standard deviation (SD). Non-parametric Mann–Whitney *U* test was for (*A*). Unpaired two-tailed Student’s *t* test with or without Welch’s correction analysis for (*A*, *C* and *D*). Statistical significance: ∗*p* < 0.05, ∗∗∗*p* < 0.01.

As shown in Figure 8, immunofluorescence (IF) staining indicated that the expression levels of adherent junctions’ protein epithelial cadherin (E-cadherin) and wheat germ agglutinin (WGA) stained goblets’ mucus were reduced in both small intestines and colons of *Otop2*^*−/−*^ mice compared to that of *Wt* mice (Fig. 8, *A*–*C*). Consistently corroborating these findings was Western blotting (WB) analysis which demonstrated a marked reduction in expression levels of E-cadherin within both small intestine and colon tissues from *Otop2*^*−/−*^ mice relative to those derived from their *Wt* counterparts (Fig. 9*A*). Moreover, immunofluorescencee (IF) staining showed the expression of tight junctions’ protein zonula occludens-1 (ZO-1) was decreased in intestinal epithelial cells of *Otop2*^*−/−*^ mice compared to that of *Wt* counterparts (Fig. S13, *A* and *B*). Transmission electron microscopy (TEM) analysis unveiled damage to intercellular junctions among intestinal epithelial cells along with irregular microvilli distribution in *Otop2*^*−/−*^ mice when juxtaposed with their *Wt* littermates (Fig. 9*B*). In addition, we noted a significant impairment in crypt proliferation within *Otop2*^*−/−*^ mice *versus* their *Wt* littermates. IHC staining revealed a marked reduction in the presence of proliferative marker Ki67-positive cells within the mucosal crypts of *Otop2*^*−/−*^ mice (Fig. S14, *A* and *B*). WB analysis first showed the proliferating cell nuclear antigen (PCNA) and Cyclin D1 reduced in intestines of *Otop2*^*−/−*^ mice compared to their control littermates (Fig. 9*C*). To further elucidate the impact of *Otop2* on crypt growth, we isolated crypts and cultured organoids derived from the mucosa of both of *Otop2*^*−/−*^ mice and their *Wt* littermates. As illustrated in Figure 9*D*, there was a conspicuous decrease in organoid budding among *Otop2*^*−/−*^ specimens compared to controls (Fig. 9*D*).

### Otop2 deficiency impaired autophagy within Paneth cells

**Figure 10:**
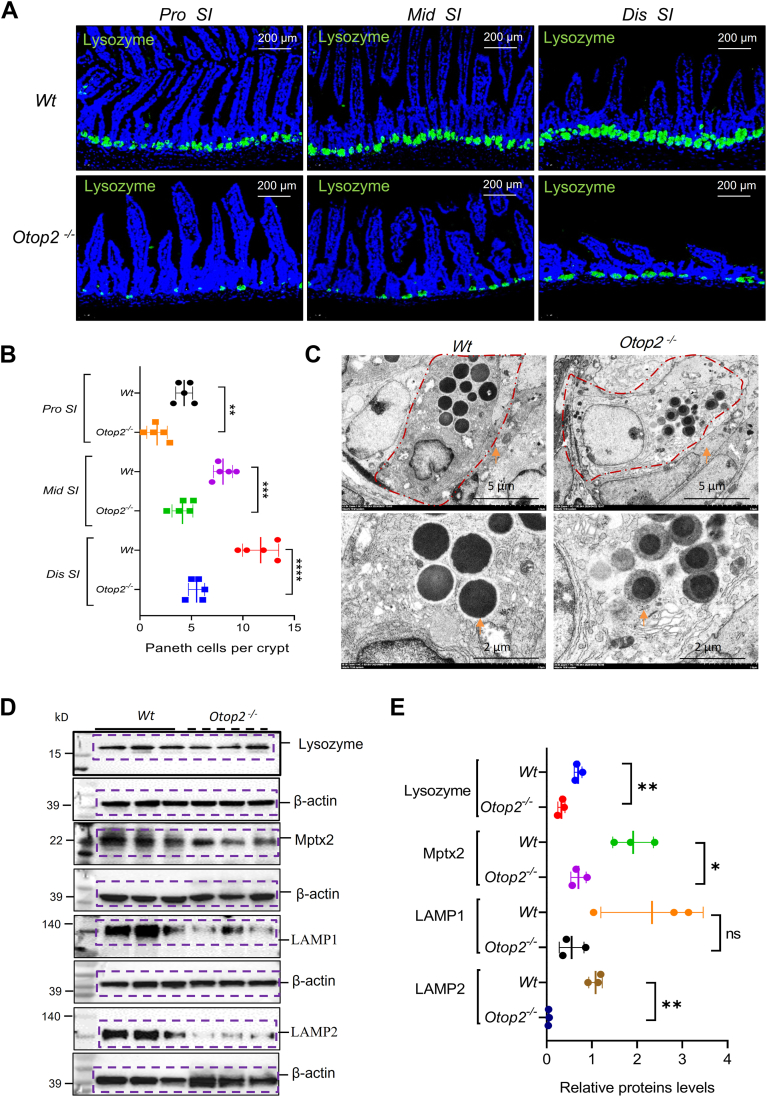
**Loss of *Otop2* decreased lysozyme in the Paneth cells.***A*, representative images of immunofluorescence (IF) staining of lysozyme for the proximal (pro), middle (mid), and distal (dis) small bowel from both *Otop2*^*−/−*^ mice (n = 5) and *Wt* mice (n = 5); Lysosome (*green*) and 4′,6-diamidino-2-phenylindole (DAPI, *blue*). *B*, quantification of lysozyme-positive cells in panel (*A*). *C*, transmission electron microscopy (TEM) analysis for Paneth cells. Arrows indicated granules. *D*, Western blotting (WB) analysis for Lysozyme, mucosal pentraxin 2 (Mptx2), Lysosomal-Associated Membrane Protein 1 (LAMP1), and Lysosomal-Associated Membrane Protein 2 (LAMP2) in mucosa of small bowel and colon from *Otop2*^*−/−*^ mice and *Wt* mice (each group, n = 3). *E*, Quantification of proteins levels in panel (D). Data presented in (*B* and *E*) was expressed as the mean ± standard deviation (SD). Unpaired two-tailed Student’s *t* test with or without Welch’s correction analysis for (*B*) and (*E*). Statistical significance: ns, not significant, ∗*p* < 0.05, ∗∗*p* < 0.01, ∗∗∗*p* < 0.001.

**Figure 11:**
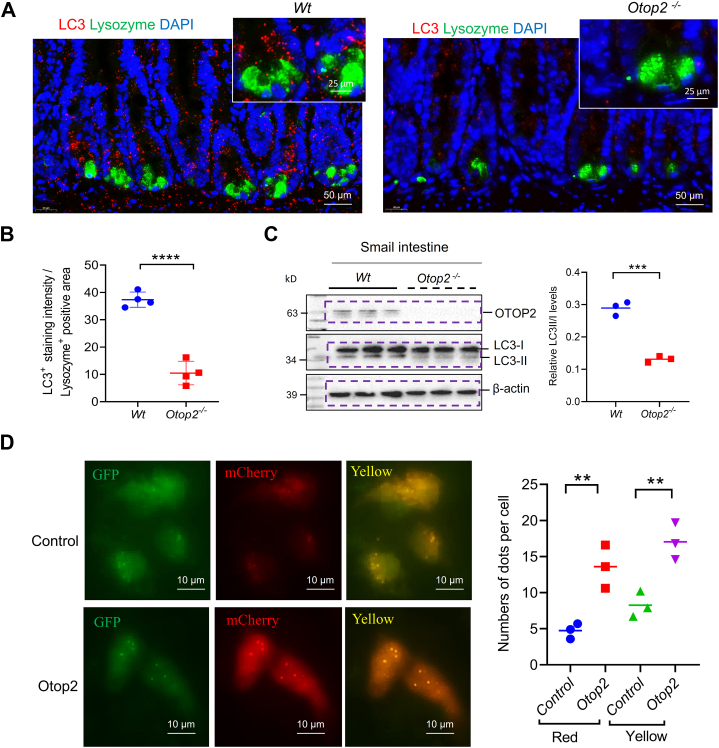
***Otop2* deficiency impaired the autophagy in Paneth cells.***A*, representative images of Immunofluorescence analysis for microtubule-associated protein 1A/1B-light chain 3 (LC3) and Lysosome in the sections of small intestine of *Otop2*^*−/−*^ mice and *Wt* mice. *B*, quantification of LC3 and Lysozyme positive cells in panel (*A*) (each group, n = 4). *C*, the Western blotting (WB) analysis was used to determine the expression levels of OTOP2 and LC3 in the small intestinal mucosa of *Otop2*^*−/−*^ mice and *Wt* mice. Quantification of them against β-actin (each group, n = 3). Independent experiments at least two times. *D*, U2OS cells infected with Ad-mCherry-GFP-LC3B adenovirus and representative images of LC3 dots with or without expressing *Otop2*. The number of *red* and *yellow* LC3 dots per cell were counted. Data presented in (*B*, *C* and *D*) was expressed as the mean ± standard deviation (SD). Unpaired two-tailed Student’s *t* test with or without Welch’s correction analysis for (*B*, *C*, and *D*). Statistical significance: ∗∗*p* < 0.01, ∗∗∗*p* < 0.001, ∗∗∗∗*p* < 0.0001.

Immunofluorescence (IF) staining-based detection of lysozyme-expressing cells revealed a marked reduction in the count of Paneth cells (PCs) within the small-intestinal mucosa of *Otop2*^*−/−*^ mice when juxtaposed with their *Wt* littermates (Fig. 10, *A* and *B*). Representative electron microscopy images further illustrated that *Otop2*^*−/−*^ mice exhibited fewer and smaller granules (Fig. 10*C*). Moreover, WB analysis demonstrated a decline in key markers associated with PCs, including Lysozyme and Mptx2, alongside lysosomal markers LAMP1 and LAMP2 in the intestinal mucosa of *Otop2*^*−/−*^ mice (Fig. 10, *D* and *E*). *MAP1LC3 (LC3),* recognized as an autophagy marker capable of capturing and eliminating invading bacteria (35), was also assessed. IF staining indicated that both the number of LC3 puncta and instances of LC3/Lysozyme colocalization were significantly diminished in the intestinal mucosa of in *Otop2*^*−/−*^ mice compared to those of *Wt* mice (Fig. 11, *A* and *B*). WB analysis confirmed that expression of LC3-II was significantly reduced in the intestinal mucosa of *Otop2*^*−/−*^ mice compared to those of *Wt* mice (Fig. 11*C*). We further transfected U2OS cells with Ad-mCherry-GFP-LC3B adenovirus to monitor the autophagy flux in the presence of *Otop2*. As shown in Figure 11*D*, both yellow and red dots increased after *Otop2* overexpression. Owing to the fact that yellow dots (merged by mCherry and GFP fluorescence) indicate the autophagosomes that are not fused with lysosome, while red dots (mCherry fluorescense) indicate the compartments that have been fused with lysosome (Fig. 11*D*). Thus, the increase of both red and yellow dots indicates the activation of autophagy, suggested that *Otop2* is essential to autophagy in PCs.

### Otop2 deficiency suppressed phagocytosis capacity of macrophages

**Figure 12:**
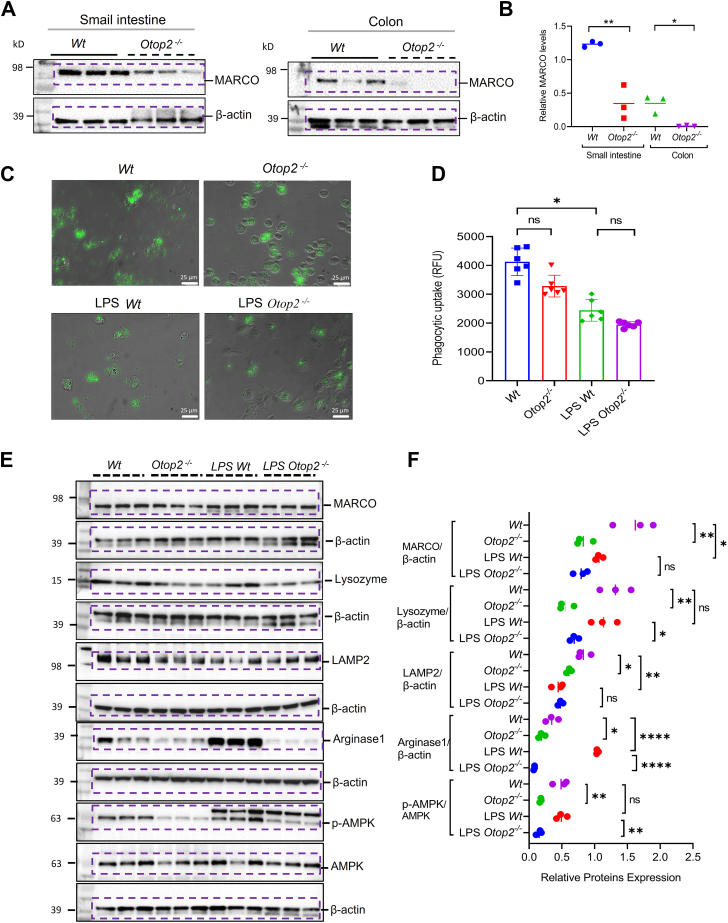
***Otop2* deficiency attenuated phagocytosis-lysozymal function of bone marrow-derived macrophages (BMDMs).***A*, representative images of Western blotting (WB) analysis for macrophage receptor with collagenous structure (MARCO) in small intestines and colon from both *Otop2* knockout (*Otop2*^*−/−*^) mice and *Wild type* (*Wt)* mice (each group, n = 3). *B*, quantification of proteins expression in panel (*A*). *C*, \Representative images of phagocytic uptake of latex beads by bone marrow-derived macrophages (BMDMs) from *Otop2*^*−/−*^ mice (n = 6) and *Wt* mice (n = 6). *D*, quantification of average fluorescence intensity of intracellular latex beads in panel (*C*). *E*, representative images of WB analysis for MARCO, Lysozyme, LAMP2, phosphorylation Signal Transducer and Activator of Transcription 3 (p-STAT3), Arginase1 (ARG1), and phosphorylation AMP-activated protein kinase (p-AMPK) in the BMDMs from both *Otop2*^*−/−*^ mice and *Wt* mice with or without lipopolysaccharide (LPS)-stimulation (each group, n = 3). *F*, quantification of proteins expression in panel (*E*). Data presented in (*B*, *D* and *F*) was expressed as the mean ± standard deviation (SD). Unpaired two-tailed Student’s *t* test with or without Welch’s correction analysis for (*B*); Kruskal-Wallis test followed by Dunn's multiple comparisons test was for (*D*). One-way ANOVA followed by Tukey's multiple comparisons test was used for statistical analysis (*F*). Statistical significance: ns, not significant (*p* ≥ 0.05); ∗*p* < 0.05; ∗∗*p* < 0.01, ∗∗∗∗*p* < 0.001.

In a concerted effort to elucidate the effects of *OTOP2* within the macrophages, we firstly showed that the expression of scavenger receptors *MARCO* was reduced significantly in both small intestines and colons from *Otop2*^*−/−*^ mice compared to that of *Wt* mice (Fig. 12, *A* and *B*). We next conducted a phagocytosis analysis on bone marrow-derived macrophages (BMDMs) obtained from both *Wt* and *Otop2*^*−/−*^ mice, which was conducted under conditions both devoid of and in response to lipopolysaccharide (LPS). Our initial findings revealed *Otop2* knockout resulted in a notable decrease in the number of intracellular beads and a lower level of intracellular fluorescence intensity, thereby inhibiting the phagocytic activity of BMDMs (Fig. 12, *C* and *D*). Furthermore, *Otop2* deficiency also impaired phagocytic activity of BMDMs under LPS treatment, but it could not reach significant levels (Fig. 12, *C* and *D*). Consistent with this finding, *Otop2* deficiency inhibited the expression of phagocytosis receptor MARCO, along with the lysosomal maker’s lysozyme and LAMP2 (Fig. 12, *E* and *F*). *Otop2* knockout inhibited anti-inflammatory macrophage marker Arginase1 (ARG1) within BMDMs (Fig. 12, *E* and *F*). Furthermore, it was observed that the activation of energy-sensor AMPK was attenuated by *Otop2* knockout (Fig. 12, *E* and *F*).

### Loss of Otop2 aggravated dextran sulfate sodium (DSS)-induced colitis in mice

**Figure 13:**
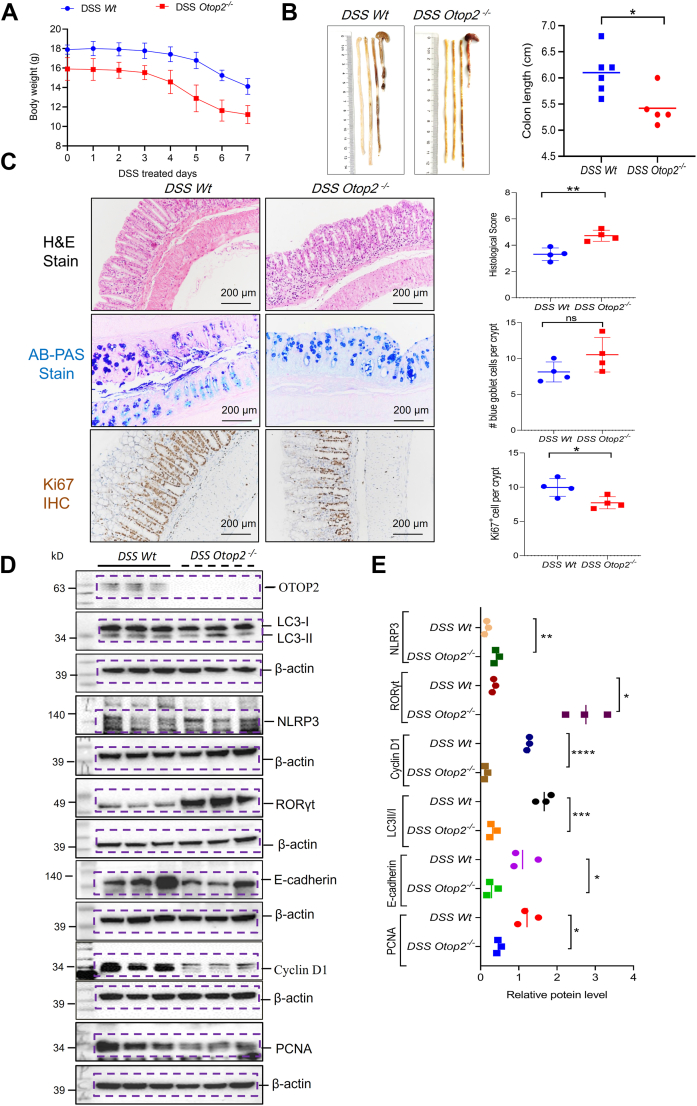
***Otop2* deficiency exaggerated dextran sulfate sodium (DSS)-induced colitis.***A*, the body weight altered between *Otop2* knockout (*Otop2*^*−/−*^, n = 5) mice and their *Wild type* (Wt, n = 5) littermates during the dextran sulfate sodium (DSS) treatment. *B*, representative images of colons from *Otop2*^*−/−*^ (n = 5) mice and *Wt*, (n = 5) mice; Quantification of length of colons of them. *C*, Representative images of hematoxylin and eosin (H&E), Alcian blue/periodic acid Schiff base (AB-PAS) staining and ki67 immunochemistry stain (IHC) in colons from both *Otop2*^*−/−*^ (n = 4) mice and *Wt*, (n = 4) mice. Quantification of them. *D*, representative images of Western blotting (WB) analysis for Otopetrin 2 (OTOP2), E-cadherin, Proliferating Cell Nuclear Antigen (PCNA), NLR Family Pyrin Domain Containing 3 (NLRP3), RAR-related Orphan Receptor Gamma t (RORγt), Cyclin D1, and Microtubule-associated protein 1A/1B-light chain 3 (LC3) proteins in colonic mucosa of *Otop2*^*−/−*^ mice and *Wt* mice with DSS treatment (each group, n = 3). (*E*) Quantification of proteins levels in panel (*D*). Data presented in (*A*, *B*, *C* and *E*) was expressed as the mean ± standard deviation (SD). Unpaired two-tailed Student’s *t* test with or without Welch’s correction analysis for (*B*, *C* and *E*). Unpaired two-tailed Student’s *t* test with or without Welch’s correction analysis for (*B*), (*C*), and (*E*). Statistical significance: ns, not significant, ∗*p* < 0.05, ∗∗*p* < 0.01, ∗∗∗*p* < 0.001, ∗∗∗∗*p* < 0.0001.

In the context of dextran sulfate sodium (DSS)-induced colitis, *Otop2*^*−/−*^ mice exhibited a more pronounced loss of body weight compared to their *Wt* counterparts (Fig. 13*A*). The lengths of the colons were significantly diminished in *Otop2*^*−/−*^ mice relative to their *Wt* littermates (Fig. 13*B*). Histopathological examination revealed that *Otop2*^*−/−*^ mice suffered from greater mucosal damage and heightened inflammatory infiltration than DSS-treated *Wt* mice (Fig. 10*C*). Notably, the number of Ki67-positive cells was markedly reduced within the mucosa of *Otop2*^*−/−*^ mice (Fig. 13*C*). Consistent with these histological observations, there was an upregulation in the expression levels of inflammatory proteins, including NLRP3 and IL-17 regulator RORγt, in the colonic mucosa of *Otop2*^*−/−*^ mice following DSS treatment when juxtaposed with their DSS-treated *Wt* littermates (Fig. 13, *D* and *E*). Furthermore, WB analysis showed that *Otop2*^*−/−*^ mice displayed diminished expression levels of tight junction protein E-cadherin, autophagy marker LC3, and proliferative protein PCNA and Cyclin D1 within their colons under conditions of DSS treatment compared to *Wt* mice (Fig. 13, *D* and *E*).

## Discussion

To the best of our knowledge, our study provides the first evidence implicating *OTOP2* depletion in the pathogenesis of IBD. We demonstrated that OTOP2 protein is enriched in Paneth cells and intestinal macrophages, and its expression is significantly reduced in pediatric IBD patients as well as in murine models of colitis induced by LPS or DSS. Functionally, *Otop2* deficiency in mice led to a systemic phenotype of growth retardation, characterized by reduced body weight, shortened intestines, and splenomegaly, and critically, rendered them markedly more susceptible to intestinal inflammation. This heightened susceptibility appears to be mediated through a multifactorial mechanism involving dysbiosis, altered luminal pH, lysosomal dysfunction in key innate immune cells, and a consequent breakdown in intestinal bacterial defense and homeostasis. Collectively, these findings position *OTOP2* as a crucial regulator at the interface of mucosal immunity, microbial ecology, and cellular metabolism.

In the human gastrointestinal tract, pH is a key factor in maintaining gut homeostasis (36). The maintenance of a precise spatial pH gradient is fundamental to gastrointestinal physiology. As noted, the luminal pH shifts from highly acidic in the stomach to a near-neutral range in the small intestine (∼6.1–7.5) and settles at a slightly acidic to neutral milieu in the colon (∼6.0–7.0) (27, 37). A key finding that elucidates the central role of *OTOP2* in gut homeostasis lies in its distinctive biophysical profile. Unlike its family members *OTOP1* and *OTOP3*, which are sharply activated only under strongly acidic conditions (thresholds < pH 6.0 and < pH 5.5, respectively), OTOP2 exhibits broad proton channel activity across an extensive pH spectrum (from pH 5 to pH 10) (38). This observation suggests that *OTOP2* is uniquely equipped for sensing luminal pH within the intestines and may play a pivotal role in maintaining intestinal homeostasis. Our study identifies the proton channel *OTOP2* as a novel and critical factor in maintaining intestinal homeostasis, with its deficiency strongly implicated in the pathogenesis of IBD. We demonstrated a significant reduction in both mRNA and protein levels of *OTOP2* within the inflamed mucosa of pediatric patients with IBD. Complementary *in vivo* studies in *Otop2*-deficient mice confirmed a direct causative role, revealing a phenotype characterized by disrupted mucosal integrity and heightened susceptibility to inflammation. Fluctuations in pH levels may indeed modulate barrier functionality and influence microbiota composition (36). Notably, the knockdown of *Otop2* expression precipitated abnormalities within both the mucus layer and tight junctions, thereby permitting bacterial infiltration into the epithelium. 16S rRNA sequencing revealed that the absence of *Otop2* significantly altered bacterial composition, leading to a reduction in bacteria from the phylum *Verrucomicrobia*-known for its association with acid metabolites in fecal samples (39), while concurrently promoting an increase in adherent-invasive *Prevotella* and Ruminococcaceae. Furthermore, scanning electron microscopy (SEM) illustrated that *Otop2*^*−/−*^ mice exhibited a heightened presence of invasive bacteria adhering to and aggregating upon the intestinal epithelial surface. Our findings both align with and extend the existing literature on pH regulation in the gut. The association between altered luminal pH and IBD is well-established, though often considered a secondary consequence (27). Our work positions OTOP2 as a primary molecular regulator of this parameter. While other proton transporters (*e.g.*, NHE3 (40)) are known to influence intestinal pH, the specific enrichment of *OTOP2* in pathways related to granule-based antimicrobial defense suggests a non-redundant, specialized role. Interestingly, some studies report decreased luminal acidity in active colitis, seemingly contradicting our finding of increased acidity in *Otop2*-deficient mice. This apparent contradiction likely underscores the complex, spatiotemporal dynamics of pH regulation. Our model likely captures an early, cell-autonomous defect (excess mucus/acidity due to failed proton import), which in the context of a prolonged, complex human disease evolves into a more variable luminal environment due to compensatory mechanisms, fluid secretion, and changes in microbial fermentation. Our findings identify OTOP2 deficiency as a key initiator of pH dysregulation and immune compromise in the gut, thereby providing a compelling mechanistic basis for the development of novel, pH-focused therapeutic strategies in IBD.

Single-cell sequencing analysis, corroborated by protein localization studies, identifies *OTOP2* as a novel marker for Paneth cells (PCs), underscoring its specialized role in this critical epithelial lineage. PCs, known for their constitutive secretion of AMPs to defend the crypt, are vulnerable to dysfunction when autophagy is impaired (13, 16, 17, 41, 42). Our findings reveal that *Otop2* deficiency directly compromises PC autophagy, leading to a significant reduction in PC numbers and a subsequent failure in AMP-mediated bacterial defense. Beyond antimicrobial defense, PCs are integral components of the intestinal stem cell (ISC) niche (43). This positioning suggests that PCs may constitute an essential niche for ISCs residing in crypts while modulating the regeneration processes of the intestinal epithelium. Our observation that *Otop2* deficiency inhibits crypt growth provides critical functional evidence for this hypothesis. Therefore, OTOP2’s role is twofold within PCs: it is essential for their cell-autonomous defense function (*via* autophagy/AMP secretion) and their non-cell-autonomous role in sustaining the regenerative capacity of the crypt. Its deficiency thus attacks intestinal homeostasis at both the defensive and reparative fronts. Macrophages are indispensable sentinels for intestinal homeostasis, reliant on a two-step bactericidal process: initial phagocytosis followed by lysosomal degradation (19, 44). The efficacy of this entire cascade is critically dependent on the latter step, as defective lysosomal degradation is known to attenuate further phagocytic capacity through a process known as "phagocytic exhaustion” (45). A key prerequisite for functional lysosomal degradation is the establishment and maintenance of a low intralysosomal pH, which activates hydrolytic enzymes like lysozyme (46). Our data reveal that *OTOP2* is a crucial molecular determinant of this process. We demonstrated that Otop2 deficiency in macrophages led to a significant inhibition of phagocytosis, coupled with a reduction in lysozyme content and an exaggerated inflammatory response to LPS. This triad of defects strongly suggests that the loss of *OTOP2* disrupts the pH-dependent maturation and function of phagolysosomes. We propose that OTOP2, likely by facilitating proton influx, is essential for establishing the acidic phagolysosomal lumen required for activating lysozyme and other hydrolases. Without this acidic "trigger," phagosomes fail to mature properly, leading to the accumulation of undigested cargo, impaired bacterial killing, and the subsequent release of pro-inflammatory signals. Thus, OTOP2 mediates a "low pH-lysozyme" axis that is fundamental for enabling macrophages to effectively clear pathogenic bacteria and resolve, rather than exacerbate, intestinal inflammation. This mechanistic insight in macrophages mirrors our findings in PCs, where *Otop2* deficiency similarly disrupted granule function and antimicrobial peptide secretion. This parallel underscores a unifying theme: OTOP2 acts as a master regulator of acidic secretory organelle function across distinct innate immune cell types in the gut. In PCs, it supports the granules that secrete AMPs into the crypt lumen. In macrophages, it supports the phagolysosomes that digest internalized bacteria. Its deficiency, therefore, cripples the two major arms of intestinal bacterial defense—epithelial secretion and myeloid phagocytosis—simultaneously. This dual-cell defect provides a powerful explanation for the severe inflammatory phenotype observed in *Otop2*-deficient mice, as the intestinal barrier is breached at both its first (epithelial) and second (myeloid) lines of defense. One of limitations of our study is the use of a global *Otop2* knockout model. The observed systemic phenotypes, including growth retardation and splenomegaly, introduce ambiguity as to whether the intestinal PCs defects are a direct, cell-autonomous consequence of *Otop2* loss or a secondary effect of broader organismal stress. Future studies employing intestinal epithelial-specific (*e.g.*, Vil1-Cre), Paneth cell-specific (*e.g.*, Defa6-Cre), macrophages-specific (*e.g.*, Cx3cr1-Cre) knockout models will be essential to isolate the intrinsic role of *Otop2* within these compartments and resolve this question of causality. While our study identifies a strong association between *Otop2* deficiency, gut dysbiosis, and elevated intestinal inflammation, the causal relationship between these phenotypes remains to be determined. It is possible that the dysbiosis is a direct consequence of altered Paneth cell function or, alternatively, that inflammation drives microbial shifts. Future studies employing Fecal Microbiota Transplantation (FMT) from *Otop2*^*−/−*^ donors into germ-free mice—will be essential to test whether the *Otop2*^*−/−*^ microbiota is sufficient to transfer the inflammatory phenotype and to disentangle host-mediated from microbiota-driven effects.

### Conclusion

This study identifies the proton channel *OTOP2* as a novel pathogenic node in IBD. We demonstrate its specific enrichment in Paneth cells and macrophages, its reduction in pediatric IBD, and the severe consequences of its loss in mice, including growth defects and breached intestinal barrier function. The key mechanistic insight is that *OTOP2* likely sustains intestinal homeostasis by ensuring optimal lysosomal activity, which in turn governs both epithelial secretion and myeloid phagocytosis—two pillars of gut innate immunity. Our findings thus link a specific molecular defect to a breakdown in the pH-microbe-defense axis, offering a new conceptual framework for understanding and potentially treating a subset of IBD.

## Experimental procedures

### Generation of Otop2 knockout (KO) mice

*Otop2*^*−/−*^ mice (Δ exons 4–7) were generated *via* genome engineering mediated by clustered regularly interspaced short palindromic repeats (CRISPRs) and CRISPR-associated protein 9 (Cas9) in C57BL/6J mice by GemPharmatech. The knockout (*KO*) region contains 1208 bp coding sequence, which loss will result in disruption of protein function. Briefly, gRNA was transcribed *in vitro* first. Next, Cas9 and gRNA were microinjected into the fertilized eggs of C57BL/6J mice. Fertilized eggs were transplanted to obtain positive F0 mice which were confirmed by PCR and sequencing. A stable F1 generation mouse model was obtained by mating Positive F0 generation mice with C57BL/6J. The genotype primers for *KO* are AGAAGACAATAGCAGCTGGCAAGG, and TGCAGGTTCACAGTTCTGTGTCAG, and the PCR production size is 263 bp. Genotype primers for *Wt* are AAATACTCCCGTGTCGGTTAGTCC, CCTGGTTTTCCTCAAGTCTCAAGG, and the PCR production size is 326 bp. All procedures involving animal subjects received approval from the Institutional Animal Care and Use Committee at Xinhua Hospital School of Medicine, Shanghai Jiao Tong University (Shanghai, China; No. XHEC-C-F-2022-010). We have adhered strictly to all pertinent ethical regulations governing animal use throughout this study. "In all animal experiments involving *Wt* and *Otop2*-deficient (*Otop2*^*−/−*^) mice, littermates of the same sex and age (±1 week) were weaned and co-housed for at least 1 week prior to the start of any experimental procedure to equilibrate their gut microbiota. For each experiment (*e.g.*, baseline characterization, DSS challenge), mice were then randomly allocated into experimental groups (*e.g.*, genotype or treatment groups) using a computer-generated random number sequence. The randomization was performed by a researcher not involved in the subsequent phenotypic assessments or data analysis to ensure allocation concealment. Investigators performing the downstream measurements were blinded to the genotype and group allocation whenever possible."

### Analysis of OTOP2 expression in pediatric inflammatory bowel disease (IBD)

In the pursuit of elucidating *OTOP2* gene expression among children afflicted with Crohn’s disease (CD) or ulcerative colitis (UC), we initially harnessed public datasets from the Gene Expression Omnibus (GEO) database for comprehensive reanalysis. The datasets pertaining to CD were sourced from prior investigations and are accessible *via* GEO accession numbers GSE57945 (31) and GSE101794 (32). Specifically, dataset GSE57945 encompasses an extensive gene expression profile derived from human ileal tissues of patients diagnosed with ileal CD (n = 143) alongside non-IBD controls (n = 42). Similarly, dataset GSE101794 presents a thorough gene expression profile from human ileal tissues belonging to individuals with ileal CD (n = 198) as well as non-IBD controls (n = 50). The datasets about studies of UC could be accessible through GEO accession number GSE109142 (33). Dataset GSE109142 includes a detailed gene expression profile of rectal tissues obtained from pediatric patients suffering from UC (n = 206), juxtaposed against control samples (n = 20). To assess the diagnostic potential of *OTOP2* for both CD and UC in children, receiver operating characteristic curves (ROCs) along with area under the curve values (AUCs) were meticulously employed. Furthermore, we evaluated the relationship between *OTOP2* levels and the pediatric ulcerative colitis activity index (PUCAI), thereby investigating its correlation with disease severity. In this study, intestinal mucosa samples were procured from our hospital: specifically five cases each of pediatric CD (three male and two female) and UC (three male and two female) alongside corresponding control specimens totaling five. The protein expression levels of *OTOP2* within these samples were rigorously analyzed. Written informed consent was duly acquired from legal guardians prior to participation. Additionally, our protocol involving human subjects received ethical approval by the Faculty of Medicine's Ethics Committee at Xin Hua Hospital under reference number XHEC-D-2022-030. All procedures performed in studies involving human participants were in accordance with the Declaration of Helsinki.

### Dextran sulfate sodium (DSS)-induced colitis

In our investigation, we utilized 6-week-old (female, n = 4; male, n = 5) and their *Wt* (female, n = 8; male, n = 7) littermates for dextran sulfate sodium (DSS)–induced colitis experiments. Additionally, untreated *Otop2*^*−/−*^ (female, n = 5; male, n = 5) mice and *Wt* (female, n = 7; male, n = 8) mice were untreated as controls. The induction of acute colitis was achieved through the administration of a solution containing 3.5% DSS (36–50 kDa; MP Biomedicals) in drinking water for 7 days. To establish a comprehensive model for DSS-induced colitis and subsequent recovery phases in mice of the C57BL/6 strain, we initiated colitis by administering a concentration of 3.5% DSS for seven consecutive days followed by an observation period allowing for recovery lasting 2 weeks (day 0, female, n = 8; male, n = 7), (day 1, female, n = 5; male, n = 5), (day 3, female, n = 5; male, n = 5), (day 7, female, n = 5; male, n = 5), (recovery 1 week, female, n = 5; male, n = 5), and (recovery 2 weeks, female, n = 6; male, n = 5).

### Lipopolysaccharide (LPS)-induced intestinal inflammation

To elicit an acute systemic inflammatory response, we administered lipopolysaccharide (LPS) intraperitoneally (i.p.; 5 mg/kg; #G5032; Wuhan Servicebio Technology Co., Ltd, Wuhan, China) to C57BL/6 mice approximately 6 weeks of age. The subjects were sacrificed at various time points: 0 h (females, n = 8; males, n = 7), 3 h (females, n = 5; males, n = 5), 6 h (females, n = 5; males, n = 5), 18 h (females, n = 5; males, n = 5), 24 h (females, n = 5; males, n = 5), and finally at the extended duration of 48 h post-LPS injection. Control groups received a saline solution as a baseline comparison.

### Histological scores

Intestinal tissues were meticulously fixed in 4% paraformaldehyde (PFA) for a duration of 24 h and subsequently sectioned into 4 μm slices for hematoxylin and eosin (H&E) staining. Utilizing the National Institutes of Health (NIH) Image software (NIH), we assessed villus height and crypt depth under a microscope (Nikon). Goblet cells were enumerated and mucous secretions quantified through Alcian blue/periodic acid–Schiff (AB/PAS) staining techniques. The histological alterations within the intestinal mucosa were graded according to previously established criteria (47, 48). In summary, colon and ileum tissue sections were independently scored by two experienced investigators who were blinded to the mouse genotype and experimental group allocation. Scoring was performed according to a well-established and semi-quantitative protocol for intestinal inflammation, which evaluates parameters such as inflammatory cell infiltration and epithelial damage. Any discrepancies in scores between the two investigators were resolved by a third, senior pathologist, also blinded to the experimental conditions, whose assessment was considered final. The final score used for analysis was the consensus score or the score from the third pathologist. Epithelial scoring was delineated as follows: 0 = normal; 1 = localized loss of goblet cells; 2 = extensive loss of goblet cells; 3 = localized loss of crypts; and 4 = significant loss of crypts across larger areas. Infiltration scores adhered to this classification: 0 = normal; 1 = infiltrate surrounding the crypt base; 2 = moderate infiltration extending to the muscularis mucosae; 3 = pronounced infiltration reaching the muscularis mucosae; and finally, 4 = infiltration penetrating into the submucosal layer.

### Isolation and treatment of bone marrow-derived macrophages (BMDMs)

The bone marrow-derived macrophages (BMDMs) were isolated and induced following established protocols (49, 50, 51). In brief, both *Wt* and *Otop2* knockout (*Otop2*^*−/−*^) mice were euthanized *via* cervical dislocation and subsequently sterilized with 75% ethanol. The femur and tibia were carefully excised under aseptic conditions. Muscle tissue adhering to the bone surface was removed, and both ends of the bones were meticulously cut using sterile scissors. Bone marrow cells were harvested by flushing the marrow cavity with DMEM medium until a whitish appearance was observed. The resulting suspension was centrifuged at 1200 rpm for 5 min, after which the supernatant was discarded. BMDMs were generated from bone marrow cells cultured in DMEM supplemented with 10% fetal bovine serum, 1% penicillin-streptomycin, and 10 ng/ml GM-CSF (#315-03-20UG, Preprotech) over a period of 9 days. On day three, an equal volume of fresh medium containing GM-CSF was added to the culture. By day seven, BMDMs that strongly adhered to the dish surface became apparent; at this point, unattached cells in the culture medium were replaced with fresh media to facilitate subsequent treatments. For experimental procedures involving cell treatment, BMDMs received exposure to lipopolysaccharide (LPS) at a concentration of 100 ng/ml for 16 h before being collected for WB.

### Phagocytosis of bone marrow-derived macrophages (BMDMs)

To evaluate the phagocytic capacity of drugs, bone marrow-derived macrophages (BMDMs) were cultured for 16 h in a drug-supplemented medium. The following day, silica beads functionalized with carboxyl groups (Sigma#L4655) were suspended in the cellular growth medium and incubated with AF488 at a concentration of 10 μg/ml at 37 °C for 30 min. After six washes with phosphate-buffered saline (PBS), the beads were resuspended in fresh medium. For cell treatments, BMDMs were exposed to lipopolysaccharide (LPS) at a concentration of 100 ng/ml for 16 h (about 12–14 h). Subsequently, the macrophages were incubated with AF488-labeled silica beads at 37 °C for an additional 2 h. The culture medium was then washed to remove any extracellular beads. Finally, cells were fixed using paraformaldehyde for 15 min, followed by three washes; optical densities were measured at excitation/emission wavelengths of 490/520 nm, and fluorescence microscopy was employed to capture images.

### Transmission electron microscopy (TEM)

We meticulously prepared intestinal samples for examination *via* transmission electron microscopy (TEM) in accordance with established protocols (52, 53). In brief, tissues harvested from six-week-old *Otop2*^*−/−*^ mice alongside their *Wt* littermates were subjected to fixation using a 2.5% glutaraldehyde solution at 23 ± 2 °C. Following this initial fixation, the specimens underwent thorough washing before being post-fixed in a 1% osmium tetroxide solution within a 0.05 mol/L sodium cacodylate buffer (pH 7.4) at a chilled temperature of 4 °C for 2 h. Subsequently, the tissues were stained with saturated uranyl acetate for an extended duration of three and a half hours at RT, followed by dehydration through graded alcohol solutions and embedding in Eponate 12 resin (Ted Pella, Inc). Thin sections were then precisely cut using a diamond knife and further stained with a saturated uranyl acetate solution diluted in 50% ethanol along with lead citrate.

### Scanning electron microscopy (SEM)

We meticulously excised approximately 5 mm^2^ of gut mucosa from both *Otop2*^*−/−*^ and *Wt* littermate mice, subsequently fixing the specimens in a 2.5% glutaraldehyde solution for 16 h at a temperature of 4 °C. Following fixation, the tissues were thoroughly rinsed, subjected to dehydration using ethyl alcohol, and then dried with carbon dioxide. The samples were coated with a fine layer of gold before being examined under a Hitachi S-4800 field emission scanning electron microscope (SEM; Hitachi).

### 16S rRNA gene-sequencing

We meticulously extracted total microbial genomic DNA samples from the gut mucosa and feces utilizing a DNeasy PowerSoil Kit (QIAGEN, Inc., Venlo, the Netherlands), adhering strictly to the manufacturer’s guidelines (54). The amplification of the bacterial 16S ribosomal RNA (rRNA) gene V4–V5 region was executed through polymerase chain reaction (PCR) employing the forward primer 515F (5′-GTGCCAGCMGCCGCGGTAA-3′) alongside the reverse primer 907R (5′-CCGTCAATTCMTTTRAGTTT-3′). To facilitate multiplex sequencing, we integrated sample-specific seven-base pair barcodes into our primers. Subsequently, PCR amplicons underwent purification *via* Agencourt AMPure Beads (Beckman Coulter) and were quantified using a PicoGreen Double-stranded Deoxyribonucleic Acid (dsDNA) Assay Kit (Invitrogen, Carlsbad, CA, USA). Following individual quantification procedures, amplicons were combined in equal proportions for paired-end sequencing-2 × 300 bp-conducted on an Illumina MiSeq platform with MiSeq Reagent Kit version 3 (Illumina, Inc.), at Shanghai Personal Biotechnology Co., Ltd in Shanghai, China. For data processing of the sequencing results as previously delineated (55), we employed the Quantitative Insights into Microbial Ecology (QIIME; https://qiime.org) pipeline version 1.8.0. The sequence data were primarily analyzed utilizing QIIME along with R software version 3.2.0 from the R Foundation for Statistical Computing based in Vienna, Austria.

### RNA sequencing

RNA-Seq (RNA Sequencing) was meticulously performed by Sangon Biotech (Shanghai, China). In brief, total RNA was extracted from the intestinal mucosa of both *Wt* and *Otop2*^*−/−*^ mice, encompassing samples from the proximal small intestine, distal small intestine, and colon; each group comprised five biological replicates. The extraction utilized the Total RNA Extractor kit (B511311, Sangon), adhering strictly to the manufacturer's protocol. Subsequently, sequencing libraries were constructed employing the VAHTSTM mRNA-seq V2 Library Prep Kit in accordance with the manufacturer’s guidelines and index codes. The quality of these libraries was rigorously assessed using the Agilent Bioanalyzer 2100 system. Paired-end sequencing of the prepared libraries was executed on NovaSeq sequencers (Illumina). FastQC (version 0.11.2) served as a tool for evaluating the integrity of sequenced data. Clean reads were aligned to a reference genome utilizing HISAT2 (version 2.0) with default parameters set forth by its developers. For statistical analysis of alignment results, RSeQC (version 2.6.1) was employed effectively. Gene expression values for transcripts were computed using StringTie (version 1.3.3 b). To elucidate sample distances and differences among groups, Principal Component Analysis (PCA) and Principal Coordinates Analysis (PCoA) were conducted. Differentially expressed genes (DEGs) between two samples were identified through DESeq2 (version 1 12.4). Furthermore, functional enrichment analyses—including Gene Ontology (GO) assessments and Kyoto Encyclopedia of Genes and Genomes (KEGG) pathway evaluations—were undertaken to ascertain significant enrichments within GO terms or metabolic pathways attributed to DEGs.

### Quantitative real-time polymerase chain reaction (qRT-PCR)

Total RNA was meticulously extracted from intestinal mucosal tissues utilizing an RNeasy kit (QIAGEN) in accordance with the manufacturer’s protocol. The quantification of RNA was performed using a NanoDrop spectrophotometer (Applied Biosystems). For reverse transcription, we employed a High-Capacity Complementary DNA (cDNA) Reverse Transcription Kit (Applied Biosystems), utilizing 2 μg of RNA as the template. Subsequently, real-time PCR reactions were conducted employing a ViiA 7 Real-Time PCR System alongside the PowerUp SYBR Green Master Mix Kit (both from Applied Biosystems). The PCR reactions were incubated in a 384-well plate at 95 °C for an initial duration of 10 min, followed by 40 cycles consisting of denaturation at 95 °C for 15 s and annealing/extension at 60 °C for 1 min. Each sample was assayed in triplicate to ensure accuracy, and data normalization was achieved against the endogenous control β-actin. Relative RNA expression levels were calculated using the ^ΔΔ^Ct method. Primers, which were adapted from previous studies (56, 57, 58) and synthesized by Invitrogen (Shanghai, China), were as following: *Otop2*, AGCAGTACAAGCCCGGAAAG, GTGTTGGTCCCACTCTCAGC; *Lyz1*, GGAATGGATGGCTACCGTGG, CATGCCACCCATGCTCGAAT; *Mptx2*, GCTCTATGTTGGGAATTCGGGA, CAATCCCAGAGCCAGACTCC; *Actb*, CACTGTCGAGTCGCGTCC, CGCAGCGATATCGTCATCCA.

### Western blotting (WB)

For WB, approximately 50 mg of tissue was meticulously homogenized in 500 μl of radioimmunoprecipitation assay (RIPA) buffer (Invitrogen, Carlsbad, CA, US), enriched with a protease inhibitor cocktail (Servicebio). The protein concentration was subsequently quantified using the Bicinchoninic acid (BCA) reagent (Pierce Biotechnology [Thermo Fisher]). Following this, equal aliquots of protein were resolved on 10% NuPAGE Bis-Tris gels (Invitrogen) and transferred onto polyvinylidene difluoride (PVDF) membranes (MilliporeSigma). After blocking with 5% nonfat milk to prevent nonspecific binding, the membranes were incubated for 16 h at 4 °C with primary antibodies. Detailed information regarding the primary antibodies utilized in this study can be found in Table S1. Subsequently, the membranes underwent three washes with tris-buffered saline containing 0.1% Polysorbate 20 (TBST), followed by incubation with secondary antibodies. After conducting final washes of the membranes using TBST, we employed an Electrochemiluminescence (ECL) Reagent Kit (Pierce) for signal detection. All original WB bands are presented in the Supplementary information section.

### Immunofluorescence (IF) and immunohistochemistry (IHC) staining

Immunofluorescence (IF) assay was performed as we described previously (59). Briefly, the intestinal tissues from were immediately fixed in 4% paraformaldehyde for 24 h and went through dehydration, clearing and paraffin embedding. Sections were mounted on positively charged slides after cutting at 4 μm thick, were then incubated with xylol and descending concentrations of ethanol. After antigen retrieval, blocking was performed using 5% bovine serum albumin for 30 min at 23 ± 2°C. The primary antibodies were incubated in a humid chamber for 16 h at 4 °C. The following day, the slides were incubated with the secondary antibody for 50 min at 23 ± 2 °C away from light after washing with phosphate-buffered saline (PBS). IHC staining was performed as previously described (59). The information for primary antibodies used in IF and IHC were listed in Table S1.

### pH imaging

pHluorin imaging was conducted in HEK-293 cells that were cotransfected with pCMV-lyso-pHluorin (Addgene, #70113) alongside either a Control plasmid (pcDNA3) or the Otop2 plasmid (Addgene). The pCMV-lyso-pHluorin construct was generously provided by Christian Rosenmund (Addgene plasmid #70113; http://n2t.net/addgene:70113; RRID: Addgene_70113). All experimental procedures were executed 48 h post-transfection. The cells were stimulated with media exhibiting varying pH levels-ranging from pH 6.5, and subsequently to pH 7.4 and pH 8.5. The cells were captured through microscopy utilizing equipment from Nikon (Tokyo, Japan), and analyzed employing ImageJ Software. This study encompassed three independent experiments to ensure robust results and reproducibility.

### Autophagy flux analysis

U2OS cells were meticulously seeded in duplicate at a density of 2 × 10^4^ cells/cm^2^ and cultured for a duration of 24 h prior to the infection. Following two thorough washes, the cells were subjected to infection with Ad-mCherry-GFP-LC3B adenovirus (Beyotime Institute of Biotechnology) at a multiplicity of infection (MOI) of 100. Concurrently, they were transfected with either Control plasmid (pcDNA3) or Otop2 plasmid (Addgene) for an additional period of 24 h. Subsequently, the infected cells underwent starvation in medium supplemented with 1% FBS for 16 h. After the specified treatment regimen, autophagy flux was meticulously observed using a laser scanning confocal microscope (Leica). The evaluation of autophagy flux was conducted by quantifying the number of yellow and red puncta present within the cellular milieu.

### Crypts isolation and organoids culture from mouse intestine

The isolation of intestinal crypts from *Wt* and *Otop2*^*−/−*^ mice and the subsequent culture of organoids were executed in accordance with previously established protocols. Briefly, following the humane euthanasia of mice *via* CO_2_ asphyxiation, the small intestine was meticulously extracted. The organ was then longitudinally incised and thoroughly rinsed with cold phosphate-buffered saline (PBS). Subsequently, the small intestine was sectioned into approximately 2 mm segments and incubated in a solution of 10 mM EDTA diluted in PBS for a duration of 20 min at 4 °C. Upon removal of the EDTA solution, the tissue fragments were suspended in 10 ml of cold PBS while discarding the supernatant. The sediment was vigorously resuspended in PBS to enrich for crypts within the supernatant. This fraction underwent filtration through a 70-μm cell strainer to eliminate any impurities present. The isolated crypts were subjected to centrifugation at 150*g* for 10 min to effectively separate them from individual cells. For mouse organoid preparation, one thousand crypts per well were embedded within Matrigel (#356255, Corning) placed in 24-well plates. The Matrigel underwent polymerization for 10 min at 37 °C before being immersed in IntestiCulture organoid growth medium (#6005, STEMCELL), which comprised 10% FBS and 1% penicillin-streptomycin. Daily photographs documenting their development were captured, while refreshing the medium every two to 3 days ensured optimal growth conditions.

### Statistics and reproducibility

Numerical source data for all charts are meticulously detailed in the Methods and Figure legends. Statistical analyses were conducted utilizing GraphPad Prism 8 Software (GraphPad, San Diego, CA), employing two-tailed unpaired t-tests or Mann–Whitney U test to compare two groups. One-way ANOVA or Kruskal-Wallis test was used to analyze multiple comparisons. Each mouse was evaluated as an individual sample, ensuring a robust assessment of variability. All data were derived from a minimum of three independent experiments, with representative findings presented and expressed as the mean ± standard deviation (SD). *p* values less than 0.05 were deemed statistically significant. The significance levels were further delineated as to ∗*p* < 0.05, ∗∗*p* < 0.01, ∗∗∗*p* < 0.001, ∗∗∗∗*p* < 0.0001.

## Data availability

The raw RNA sequence data and 16S rRNA sequence data reported in this paper have been deposited in the Genome Sequence Archive (Genomics, Proteomics & Bioinformatics 2025) in National Genomics Data Center (Nucleic Acids Res 2025), China National Center for Bioinformation/Beijing Institute of Genomics, Chinese Academy of Sciences (GSA: CRA035426; 16S rRNA: CRA035825) that are publicly accessible at https://ngdc.cncb.ac.cn/gsa. Figure 1, *A*–*E* was reanalyzed using the dataset (GSE57945, GSE101794, and GSE109142). Fig. S2 was from The Human Protein Atlas. Source images for representative WB shown in figures are provided in Fig. S15. Source data, as well as statistical analysis for all graphs, are provided in the Excel file Supplementary Data 1.

## Supporting information

This article contains supporting information.

## Conflict of interest

The authors declare that they have no conflicts of interest with the contents of this article.
