# Supplementary material for: OTOP2 proton channel couples luminal pH sensing to intestinal immune homeostasis
Source: J Biol Chem. 2026 May 7;302(6):113105. doi: 10.1016/j.jbc.2026.113105 (PMC13241748; doi:10.1016/j.jbc.2026.113105)

Supplementary information

**Proton channel OTOP2 couples luminal pH sensing to intestinal immune homeostasis**

Weihui Yan, Ying Wang_,_ Hongxia Zhao, Shicheng Peng, Ying Lu, Bo Wu and Yongtao Xiao

**Supplemental Information contents:**

1. Supplementary Table 1, Page 2 - Page 3

2. Supplementary Figures 1 -15, Page 4 – Page 42

**1. Supplementary Tables**

**Supplementary Table 1 Antibody information**

| Antibody | Source | Catalog# | Application/dilution |
| --- | --- | --- | --- |
| β-actin | Santa Cruz Biotechnology, Inc. Santa Cruz CA, USA | SC-47778 | WB (1:2000) |
| E-Cadherin | Cell Signaling Technology, Inc. Danvers, MA, USA | 3195P | WB (1:1000) |
| E-Cadherin | Proteintech Group, Inc. Wuhan, China | 20874-1-AP | WB (1:2000) |
| Lysozyme | Servicebio Inc. Wuhan, China | GB11345 | WB (1:1000)  /IF(1:2000) |
| LAMP1 | DSHB, Iowa, IO, USA | G1/139-5-S | WB (1:1000) |
| LAMP2 | DSHB, Iowa, IO, USA | ABL-93-S | WB (1:1000) |
| LC3 | Cell Signaling Technology, Inc. Danvers, MA, USA | 12741S | WB (1:1000) |
| NLRP3 | Proteintech Group, Inc. Wuhan, China | 19771-1-AP | WB (1:1000) |
| RORγt | Bioss Inc, Beijing, China | bs-23110R | WB (1:1000) |
| LC3B | Abcam, Cambridge, UK | ab48394 | WB (1:1000) |
| PCNA | Bioss Inc, Beijing, China | bs-2007R | WB (1:1000) |
| Cyclin D1 | Abcam, Cambridge, UK | Ab16663 | WB (1:2000) |
| E-Cadherin | BD Biosciences | 560061 | IF (1:200) |
| WGA | Servicebio Inc. Wuhan, China | G1731 | IF (1:200) |
| LC3 | Servicebio Inc. Wuhan, China | GB13431 | IF (1:5000) |
| ZO-1 | Servicebio Inc. Wuhan, China | GB111981 | IF(1:500) |
| OTOP2 | Invitrogen, Carlsbad, CA, USA | PA5-55101 | IHC (1:200) |
| OTOP2 | Bioss Inc, Beijing, China | bs-17535R | IF (1:50)  WB (1:1000) |
| BEST4 | Abcam, Cambridge, UK | ab188823 | IF (1:200)  IHC (1:200) |
| Ki67 | Servicebio Inc. Wuhan, China | GB111141 | IHC(1:500) |
| Mptx2 | In our lab | N/A | WB (1:200) |
| MARCO | Servicebio Inc. Wuhan, China | GB114239 | WB (1:1000) |
| p-AMPK | Cell Signaling Technology, Inc. Danvers, MA, USA | #2535 | WB (1:1000) |
| AMPK | Proteintech Group, Inc. Wuhan, China | #80209-5 | WB (1:1000) |
| P-STAT3 | Cell Signaling Technology, Inc. Danvers, MA, USA | #9145 | WB (1:1000) |
| STAT3 | Proteintech Group, Inc. Wuhan, China | #80149-1 | WB (1:1000) |
| Arginase1 | Servicebio Inc. Wuhan, China | GB11285 | WB (1:1000) |

1. **Supplementary Figures**

**Supplementary Figure 1 OTOP2 expressed in the Paneth cells.**

(A) Quantitative real-time PCR (qRT-PCR) of *Otop2* mRNA in different organs of mice. 4 - 6 mice per group. Values were normalized to beta-actin *(Actb*) expression.

(B) Representative images of immunofluorescence (IF) staining for OTOP2 in mice proximal (pro), middle (mid), distal (dis) small bowel and colon (each group, n = 4).

(C) Quantification of OTOP2 positive cells in the different segments of mice intestine in panel (B).

(D) Alteration of *Otop2* mRNA, mucosal pentraxin 2 (*Mptx2*) and Lysozyme (*Lyz1*) mRNA from the embryonic stages (E12.5 – 17.5) to the postnatal time (P0 – 13.5) (each group, n = 4).

(E) Representative images of immunofluorescence (IF) staining for OTOP2 and Lysozyme in mouse proximal (pro), middle (mid), and distal (dis) small intestines.

Data presented in (A, C, and D) was expressed as the mean ± standard deviation (SD). Ordinary One-way ANOVA followed by Tukey's multiple comparisons test for (C). Statistical significance: **p* < 0.05; ns, not significant.

**
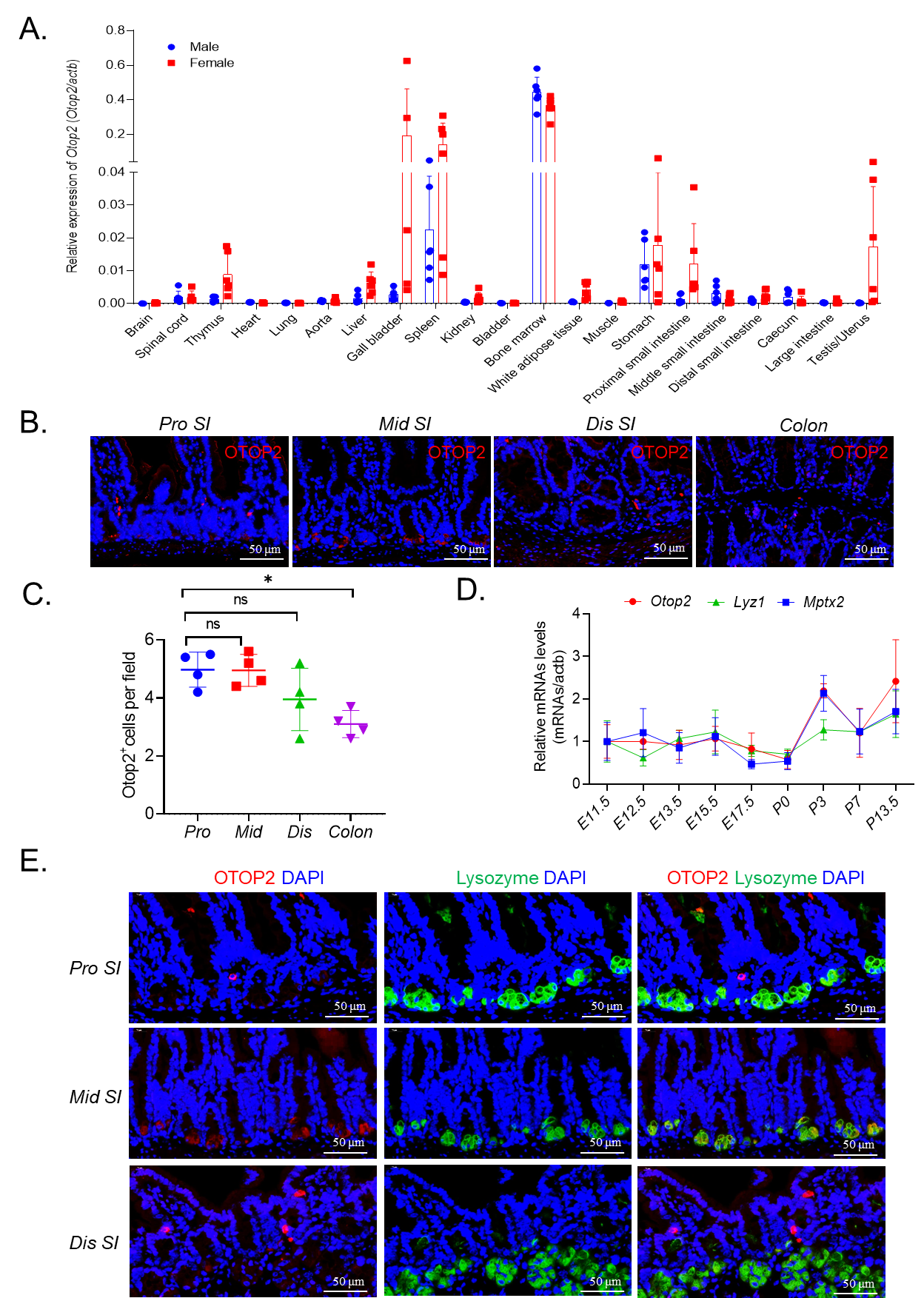
**

**Supplementary Figure 2 of OTOP2 mRNA and protein expression is enriched intestinal Paneth cells and immune cells.**

(A) RNA single cell type specificity showed OTOP2 was specially enriched in Paneth cells (https://www.proteinatlas.org/ENSG00000183034-OTOP2/single+cell/small+intestine) and MARCO positive macrophages (https://www.proteinatlas.org/ENSG00000183034-OTOP2/single+cell/colon#tissue_cell_type).

(B) Immunofluorescent stain showed OTOP2 protein located at the plasma membrane of immune cells (HPA024524: HDLM-2 cells).

**
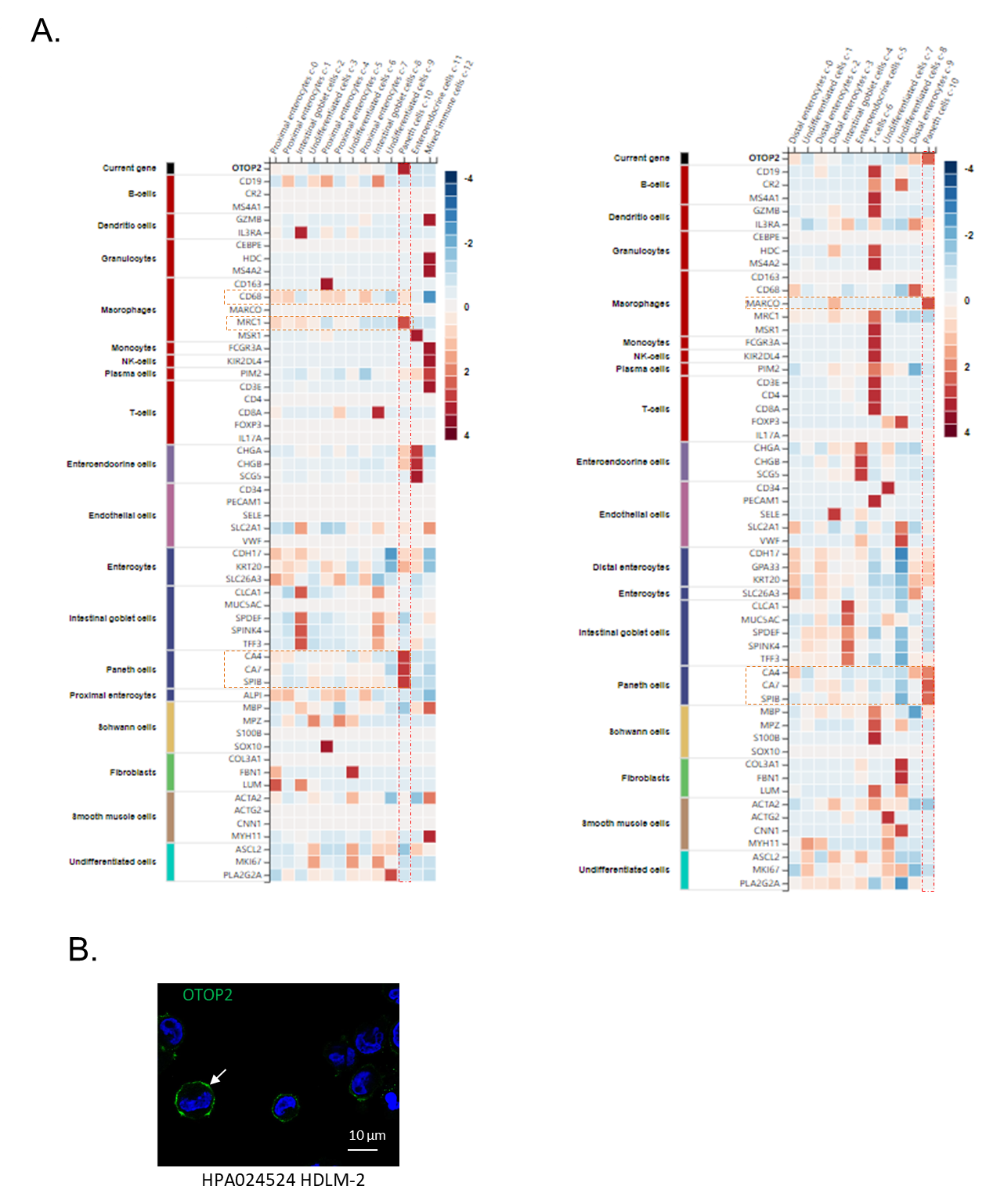
**

**Supplementary Figure 3 Decreased expression of Bestrophin 4 (BEST4) protein in diseased intestinal tissues of children with Crohn's disease (CD) and ulcerative colitis (UC).**

(A) Representative images of immunofluorescence (IF) staining for otopetrin 2 (OTOP2) and bestrophin 4 (BEST4) in uninflamed colonic mucosa from pediatric patients with ulcerative colitis (UC).

(B) Representative immunohistochemistry (IHC) images depicting the localization of Bestrophin 4 (BEST4) in the intestinal tissues of pediatric patients diagnosed with CD and UC.

(C) Quantification of BEST4 IHC staining is shown for each group, n = 5.

Data presented in (C) was expressed as the mean ± standard deviation (C). Statistical analysis was performed using an unpaired two-tailed Student’s t-test, with or without Welch’s correction, as applicable to panel (C). Statistical significance: ***P < 0.001;****P< 0.0001

**
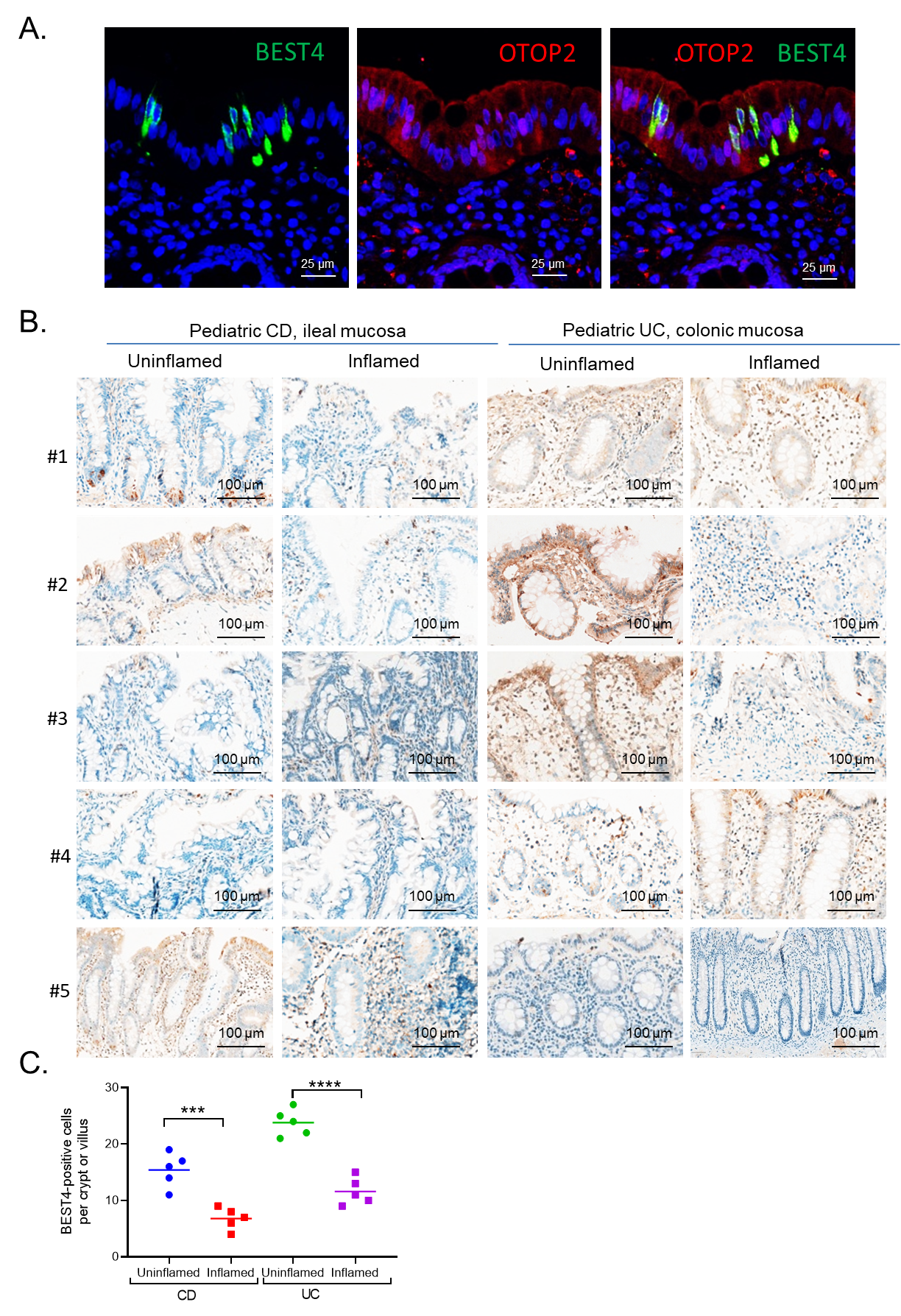
**

**Supplementary Figure 4 Expression levels of Bestrophin 4 (BEST4) are diminished in the intestinal tissues of children with Crohn's disease (CD) and ulcerative colitis (UC).**

(A) Data on BEST4 mRNA expression were extracted from the GEO database, comparing intestinal tissues from patients diagnosed with Crohn's disease (CD) or ulcerative colitis (UC) to non-inflammatory bowel disease (non-IBD) control subjects. The datasets include GSE57945, where CD samples number n=143 and controls n=42; GSE101794, with CD samples n=198 and controls n=50; and GSE109142 for UC, comprising n=206 for cases and n=20 for controls.

(B) The area under the curve (AUC) for OTOP2 was assessed in differentiating UC or CD patients from non-IBD patients. The AUC value is presented along with a 95% confidence interval.

(C) Comparison of mRNA expression levels of BEST4 between CD children with deep ulcers versus those without.

(D) Analysis of mRNA expression levels of BEST4 among UC children categorized by varying degrees of disease severity.

(E) Correlation analysis between mRNA expression levels of BEST4 and PUCAI scores in UC children was conducted using data extracted from dataset GSE109142, which included Mild-PUCAI (n=54), Moderate-PUCAI (n=83), and Severe-PUCAI groups (n=69). Data presented in (A, C, and D) was expressed as the mean ± standard deviation (SD). Non-parametric Mann–Whitney U test was for (A and C). The Kruskal-Wallis test followed by Dunn's multiple comparisons test was for (D). Statistical significance is indicated as follows: **p* < 0.05; ***p* < 0.01; *****p* < 0.0001; ns indicates not significant. Abbreviations: Ctrl, Control; PUCAI, Pediatric Ulcerative Colitis Activity Index; AUC,Area Under the Curve; CD, Crohn’s Disease

**
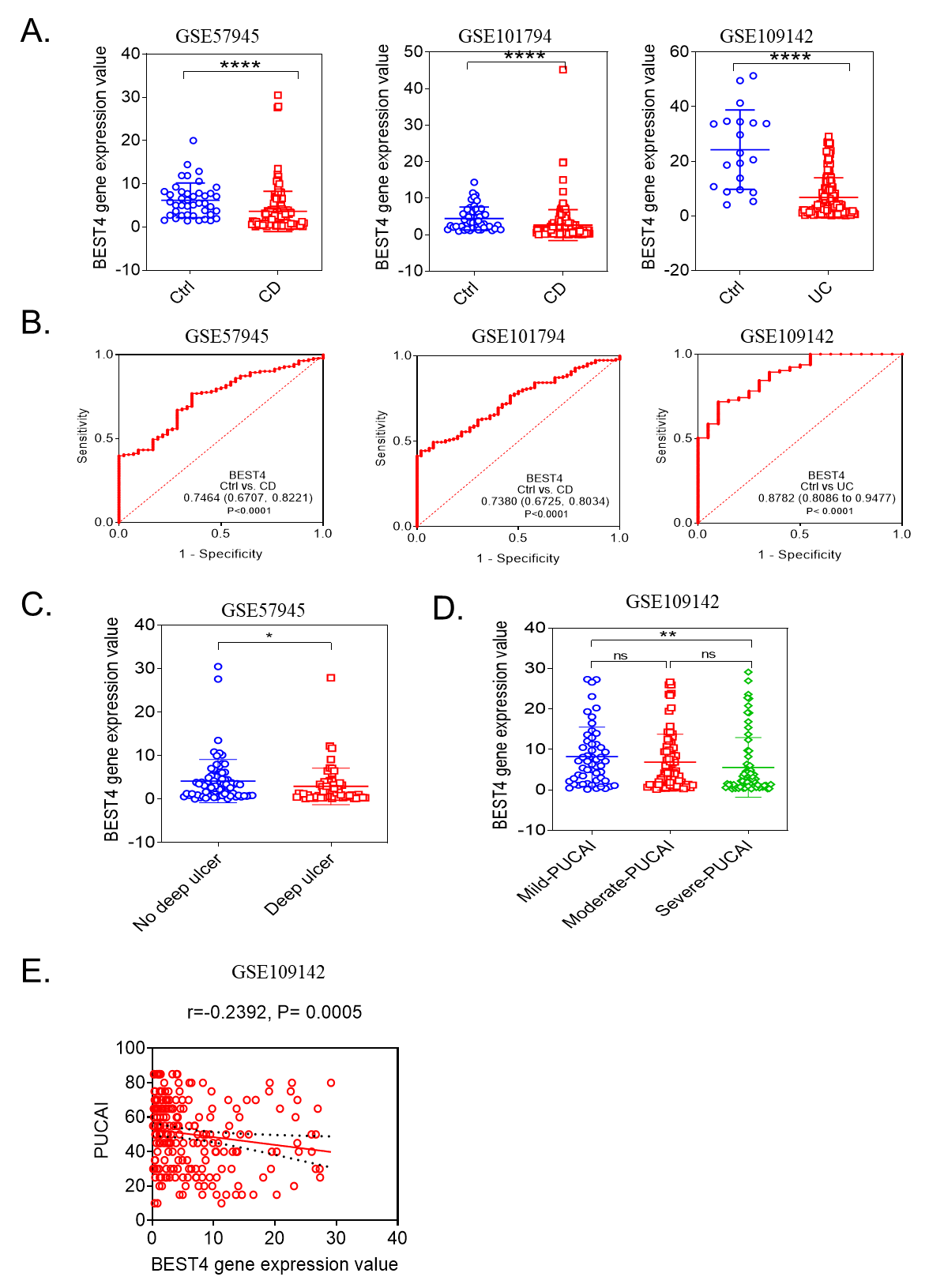
**

**Supplementary Figure 5 Generation of *Otop2* knockout (*Otop2^-/^*^-^) mice and genotyping.**

(A) The structure of *Otop2* gene, exons 4 - 7 of *Otop2* (ENSMUST00000055490.8) transcript is the knockout region. The region contains 1208 bp coding sequence. Knock out the region will result in disruption of protein function. The CRISPR/Cas9 technology to modify Otop2 gene.

(B) Reprehensive image of *Otop2* knockout mice genotype using the PCR analysis.

(C) Representative images of western blotting (WB) analysis for OTOP2 in small intestines and colon from both *Otop2^-/-^* mice and *Wt* mice (each group, n = 3).*KO*, knockout, *Wt*, wild type; -/-, *Otop2^-/^*^-^; -/+, *Otop2^-/^*^+^ ; +/+, *Wt*


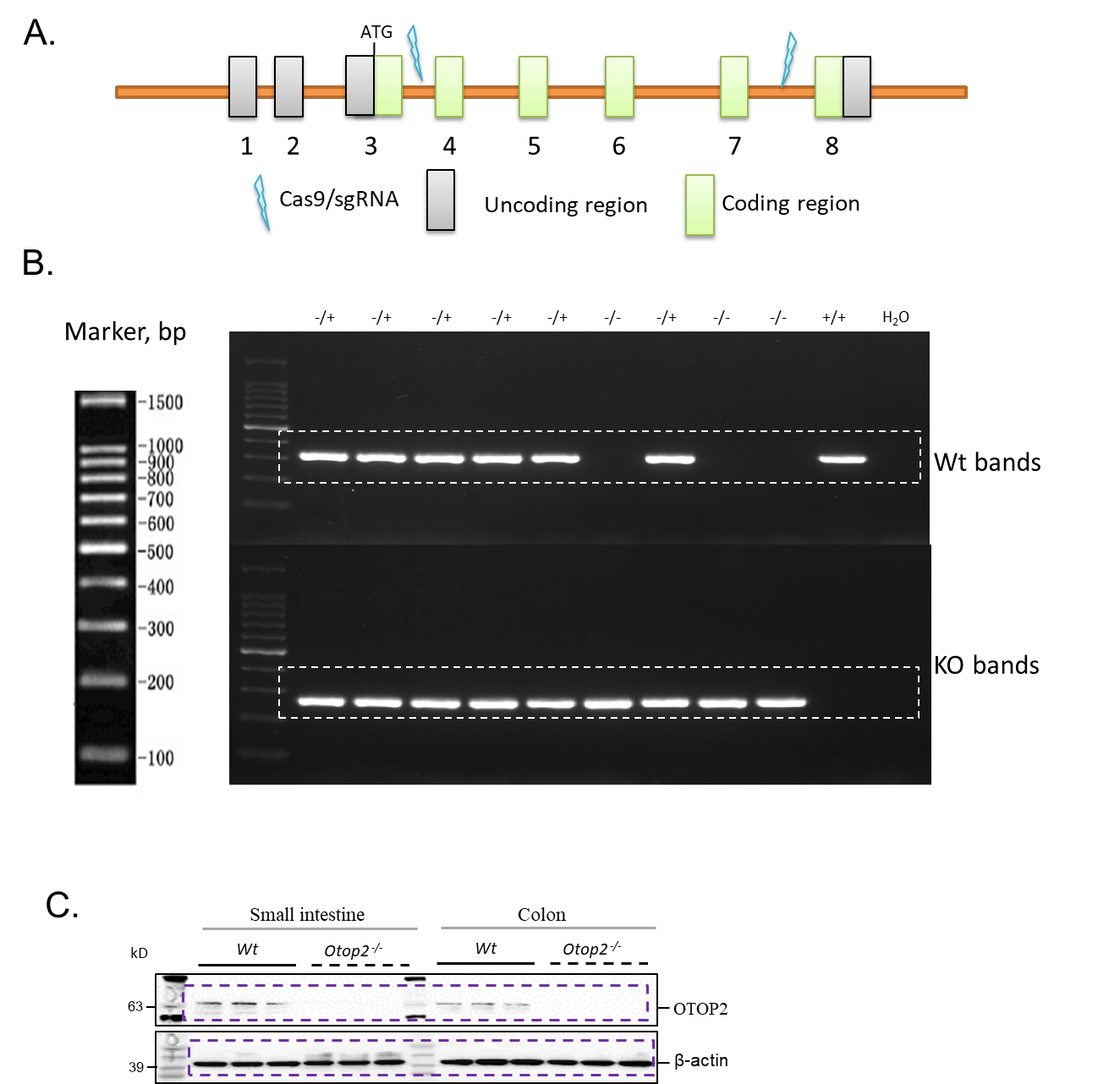


**Supplementary Figure 6 Histological alterations in organs between *Otop2* knockout (*Otop2^-/-^*) and *wild- type* (*Wt*) mice.**

(A) Representative images of brains from *Otop2^-/^*^-^; *Otop2^-/^*^+^ and, *Wt* mice*.*

(B) Representative images of histology for brain, liver, pancreas, stomach, kidney and spleen in of *Otop2^-/^*^-^; *Otop2^-/^*^+^ and, *Wt* mice.


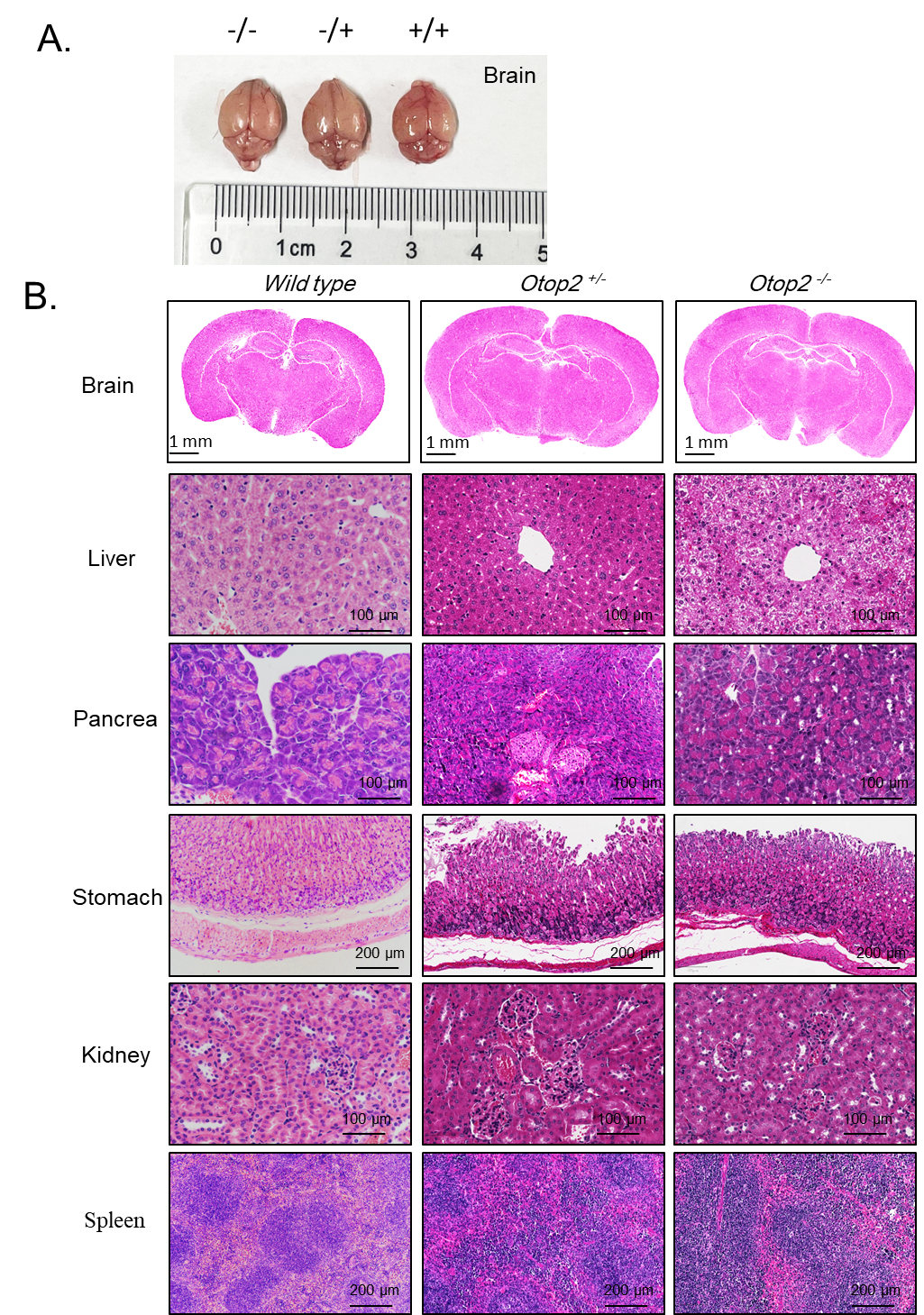


**Supplementary Figure 7 The differentially expressed genes in the distal (Dis) small intestinal mucosa of *Otop2^-/-^*** m**ice and *Wt* mice.**

(A) Heatmap of differentially expressed genes in the distal (Dis) small intestines of *Otop2^-/-^* mice (n = 3) and *Wt* mice (n = 5).

(B) Gene Ontology (GO) enrichment analysis results of differentially expressed genes in the small intestines of *Otop2^-/-^* mice and *Wt* mice.

**
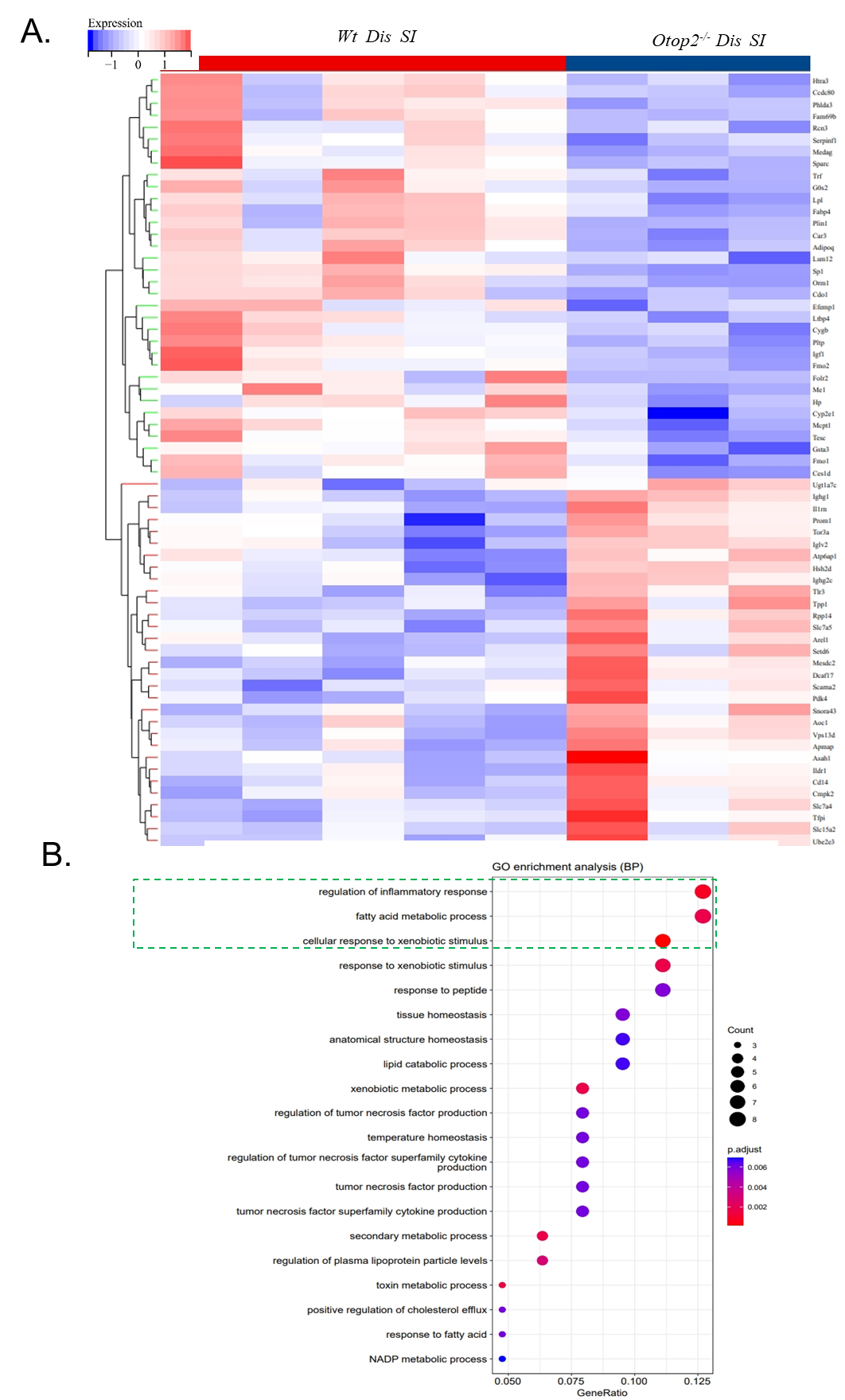
**

**Supplementary Figure 8 The differentially expressed genes in the colonic mucosa of *Otop2^-/-^*** m**ice and *Wt* mice.**

(A) Heatmap of differentially expressed genes in the colons of *Otop2^-/-^* mice (n = 3) and *Wt* mice (n = 5).

(B) Gene Ontology (GO) enrichment analysis results of differentially expressed genes in the small intestines of *Otop2^-/-^* mice and *Wt* mice.

**
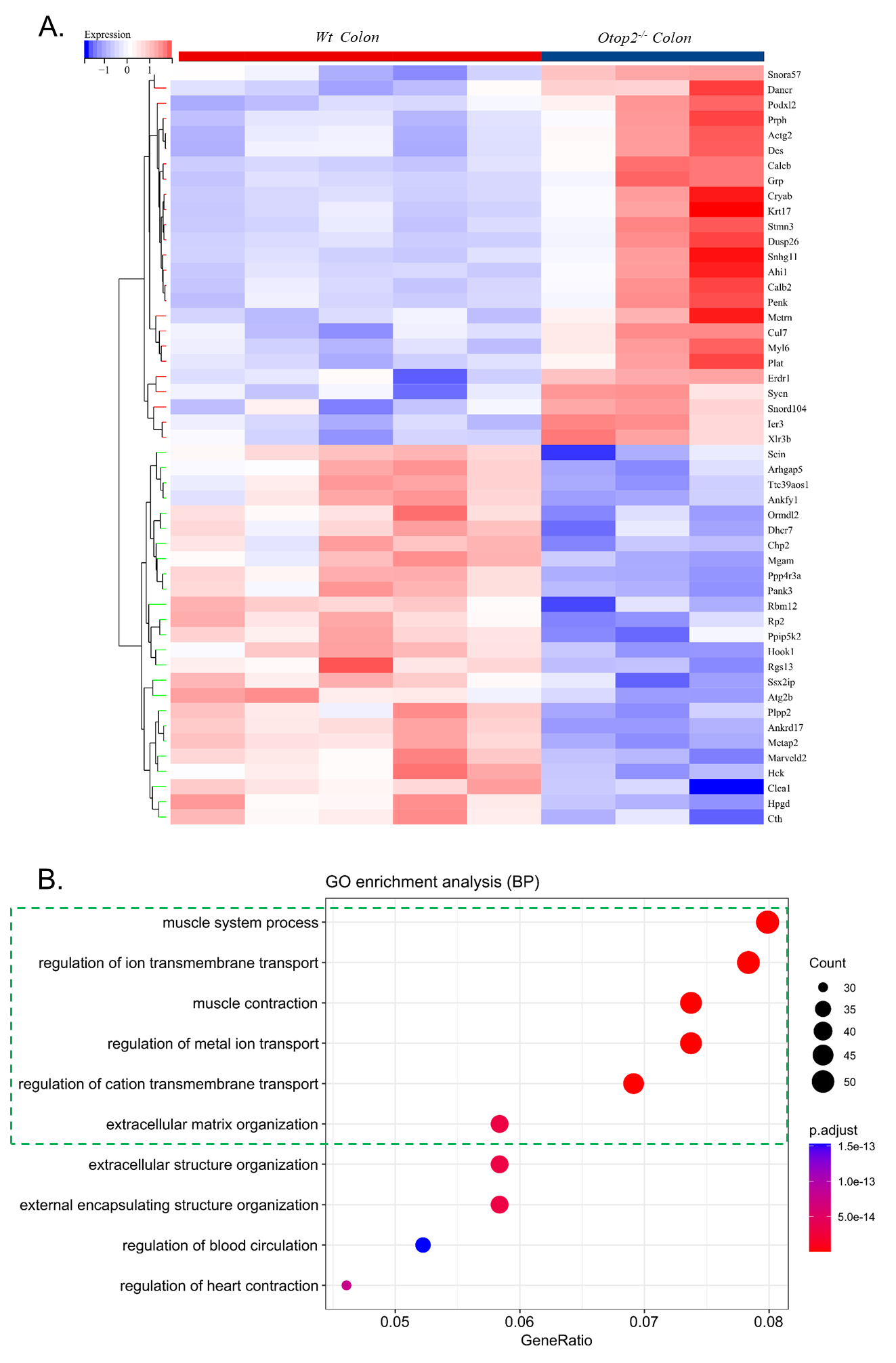
**

**Supplementary Figure 9 *Otop2* deficiency altered the microbiota composition in feces.**

(A) Principal co-ordinates analysis (PcoA for altered bacteria in the feces of *Otop2* knockout (*Otop2^-/-^*) and wild type (*Wt*) mice (Each group, n = 4 - 5).

(B) The relative abundance of the top bacteria (phylum) in the feces of *Otop2^-/-^* mice and *Wt* mice.

(C) Linear discriminant analysis Effect Size (LEfSe) analysis of the dominant altered genus in the feces of *Otop2^-/-^*mice and *Wt* mice.


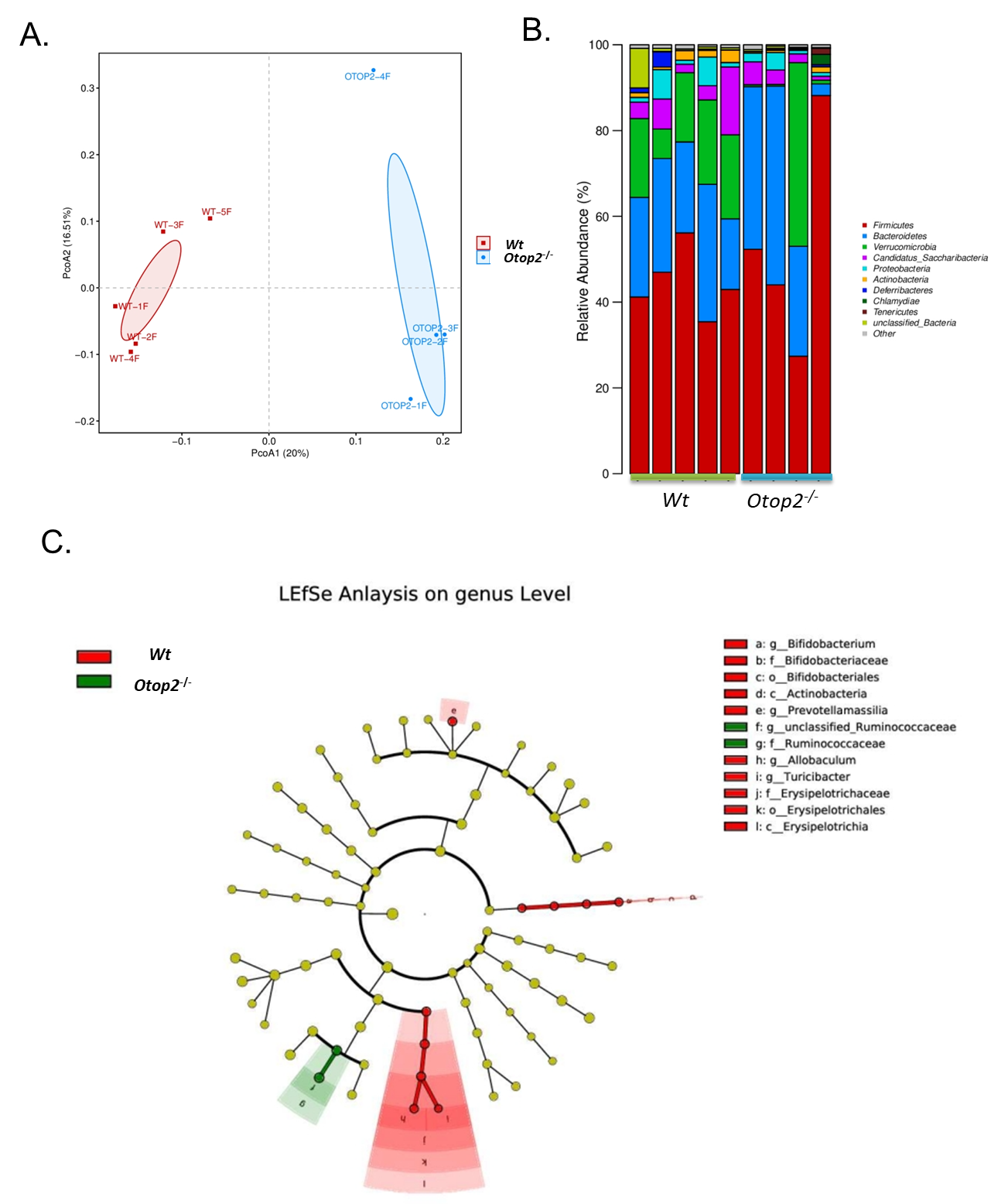


**Supplementary Figure 10. Multi-omics analysis reveals altered microbiota composition associated with gene expression changes in the proximal small intestine.** A heatmap displays Spearman’s rank correlation coefficients between differentially abundant microbiota and differentially expressed genes in the proximal small intestinal mucosa from *Otop2* knockout (*Otop2^−/−^*) and *wild-type* (*Wt*) mice. Correlations were calculated using Spearman’s rank correlation. Statistical significance is denoted as **p* < 0.05; ***p* < 0.01.

**
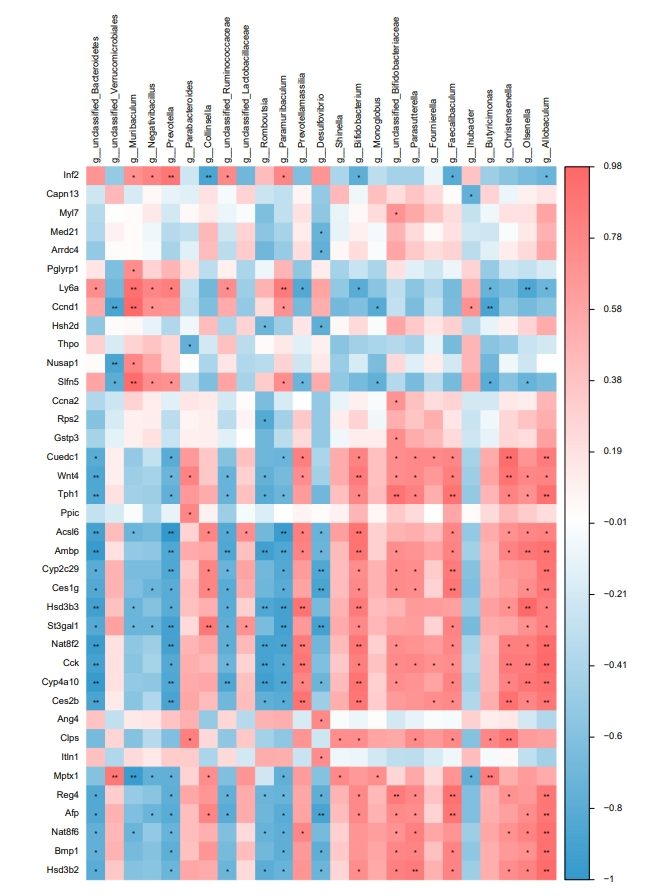
**

**Supplementary Figure 11. Multi-omics analysis reveals altered microbiota composition associated with gene expression changes in the distal small intestine.** A heatmap displays Spearman’s rank correlation coefficients between differentially abundant microbiota and differentially expressed genes in the distal small intestinal mucosa from *Otop2* knockout (*Otop2^−/−^*) and *wild-type* (*Wt*) mice. Correlations were calculated using Spearman’s rank correlation. Statistical significance is denoted as **p* < 0.05; ***p* < 0.01.

**
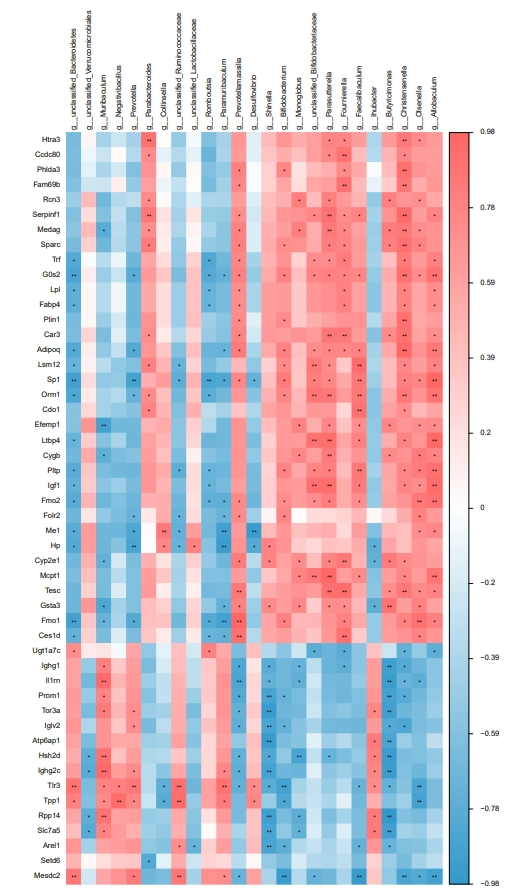
**

**Supplementary Figure 12. Multi-omics analysis reveals altered microbiota composition associated with gene expression changes in the colon.** A heatmap displays Spearman’s rank correlation coefficients between differentially abundant microbiota and differentially expressed genes in the colonic mucosa from *Otop2* knockout (*Otop2^−/−^*) and *wild-type* (*Wt*) mice. Correlations were calculated using Spearman’s rank correlation. Statistical significance is denoted as **p* < 0.05; ***p* < 0.01

**
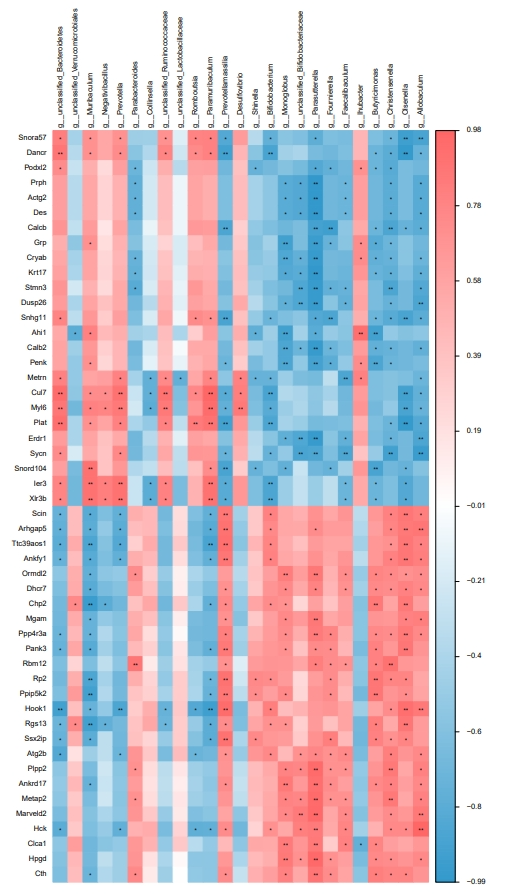
**

**Supplementary Figure 13 Tight junction protein ZO1 is altered between *Otop2* knockout (*Otop2^-/-^*) and wild type (*Wt*) mice.**

(A) Representative images of ZO1 (green) for the proximal (pro), middle (mid), distal (dis) small bowel and colon from both *Otop2^-/-^* mice (n = 3) and *Wt* mice (n = 3).

(B) Quantification of panel (A).

Data presented in (B) was expressed as the mean ± standard deviation (SD). Unpaired two-tailed Student’s t test with or without Welch’s correction analysis for (B). Statistical significance: * *p* < 0.05, *** *p* < 0.001.


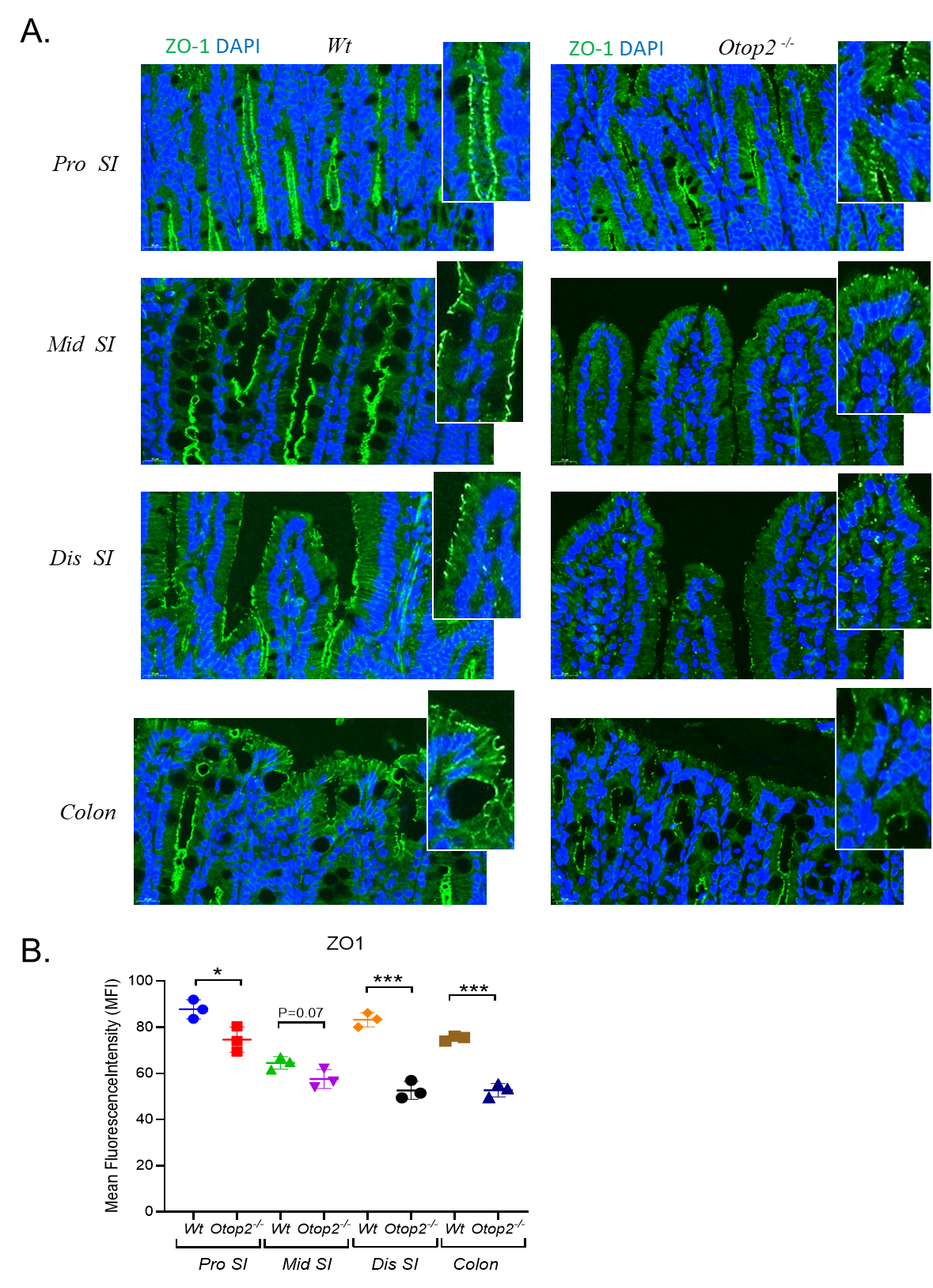


**Supplementary Figure 14 The knockout (*Otop2^-/-^*) mice intestinal growth less than that of *Wild type* (*Wt*) mice.**

(A) Representative images of ki67 immunochemistry stain (IHC) in the proximal (pro), middle (mid), distal (dis) small bowel and colon from both *Otop2^-/-^* mice (n = 4) and *Wt* mice (n = 4).

(B) Qualification of ki67-positive cells per crypt in panel (A).

Data presented in (B) was expressed as the mean ± standard deviation (SD). Unpaired two-tailed Student’s t test with or without Welch’s correction analysis for (B). Statistical significance: * *p* <0.05, ** *p* <0.01


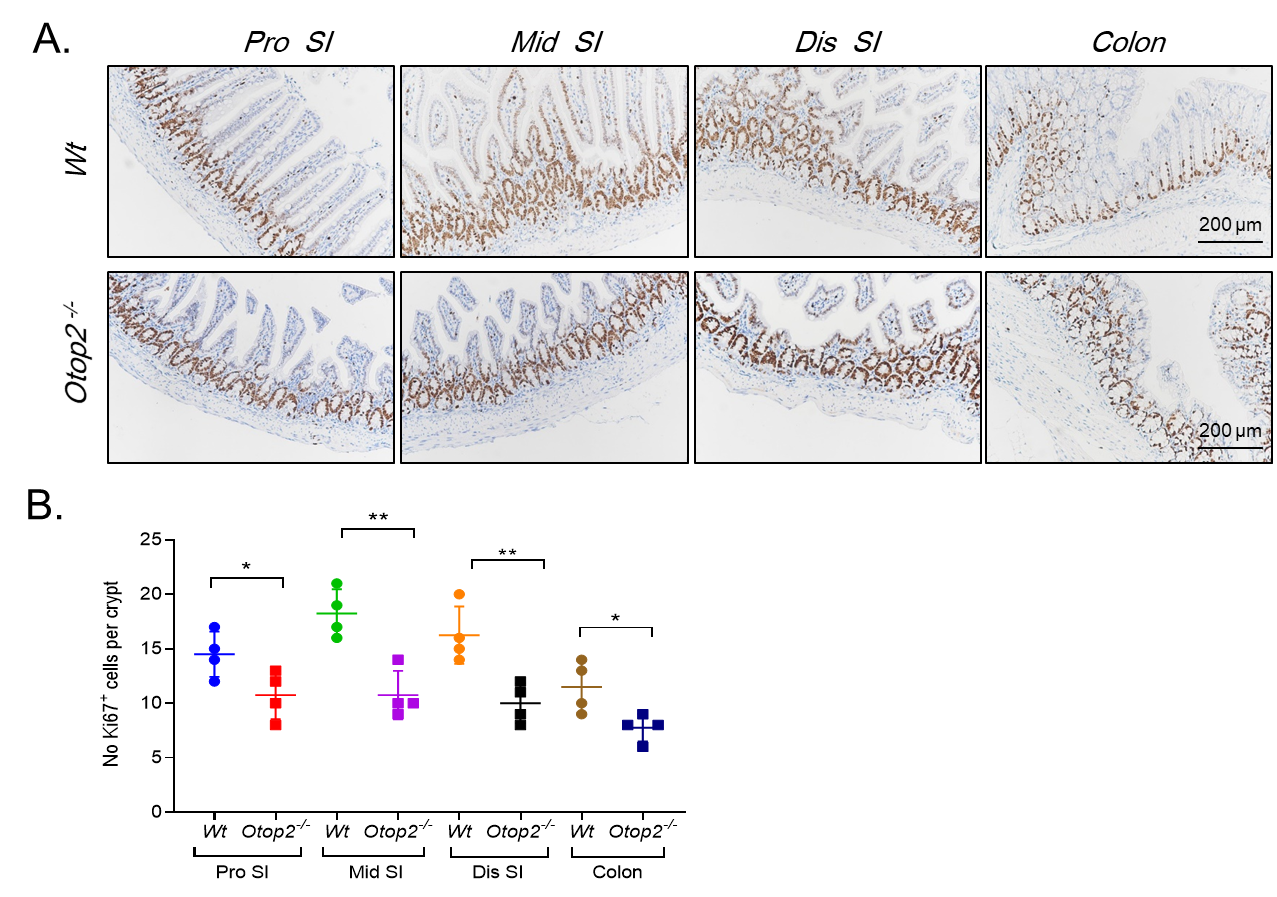


**Supplementary Figure 15 Original WB bands in Figures**

**Orignal Figure 9A**

**
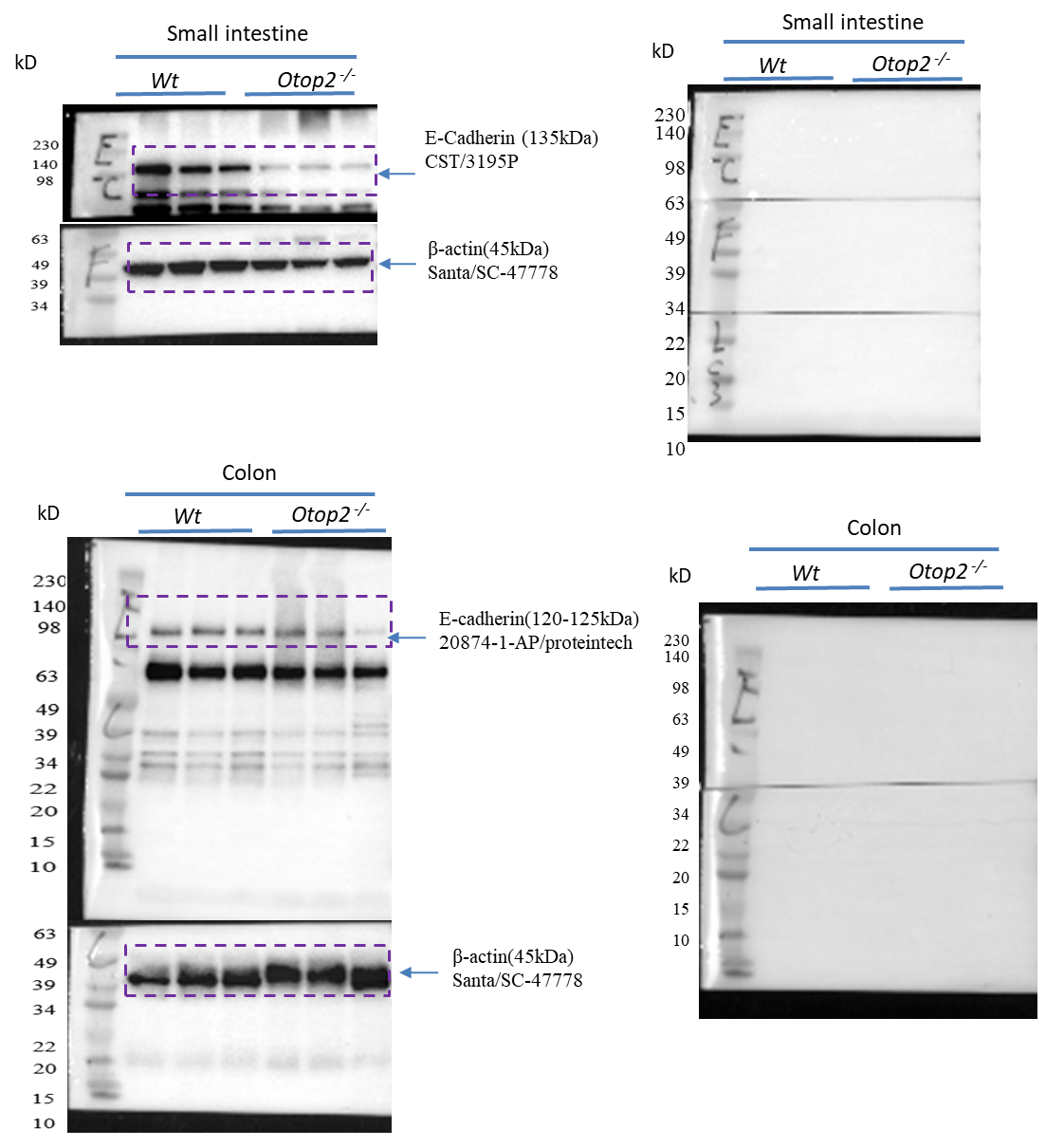
**

**Orignal Figure 9C**

**
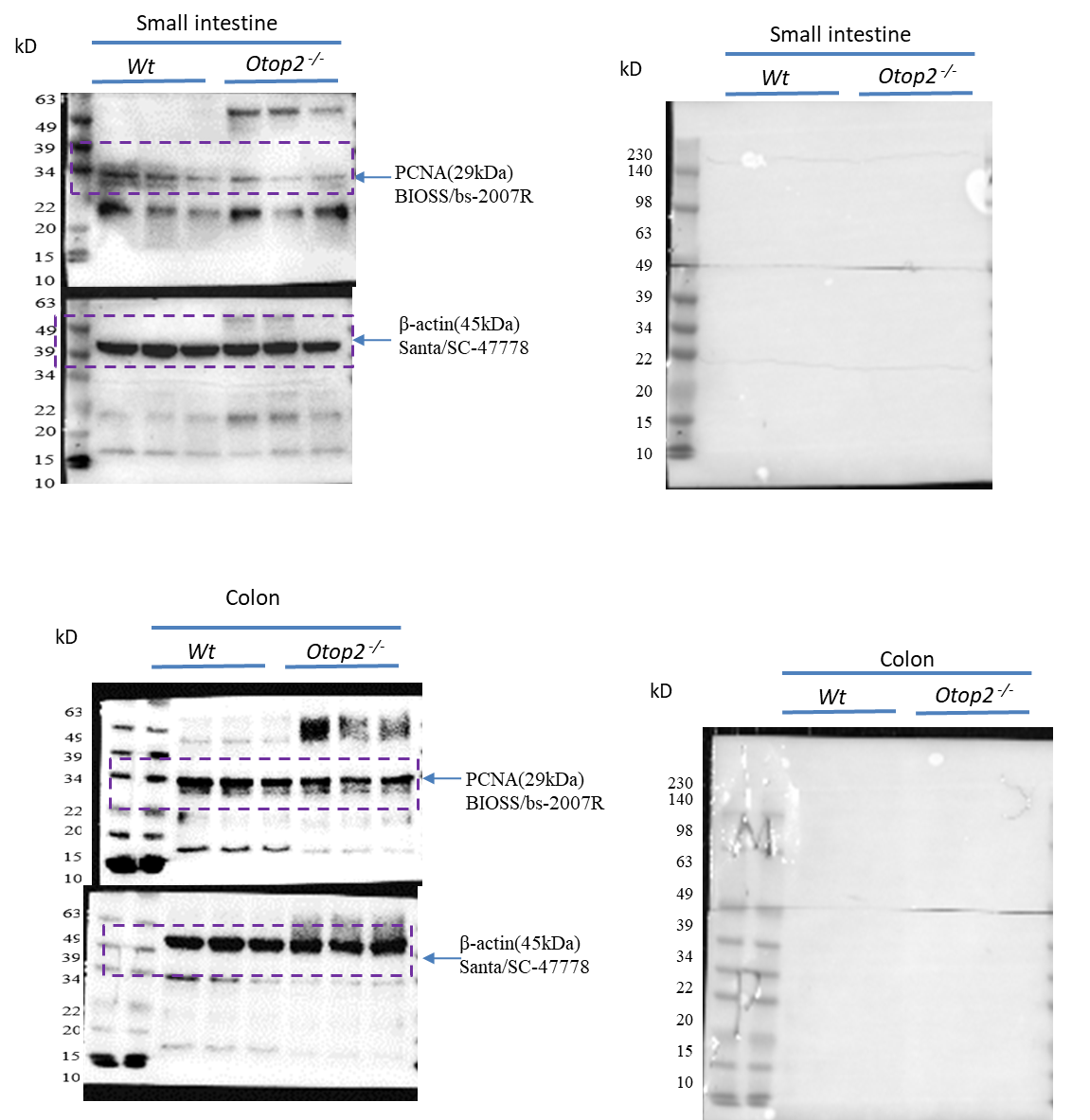
**

**Orignal Figure 9C**

**
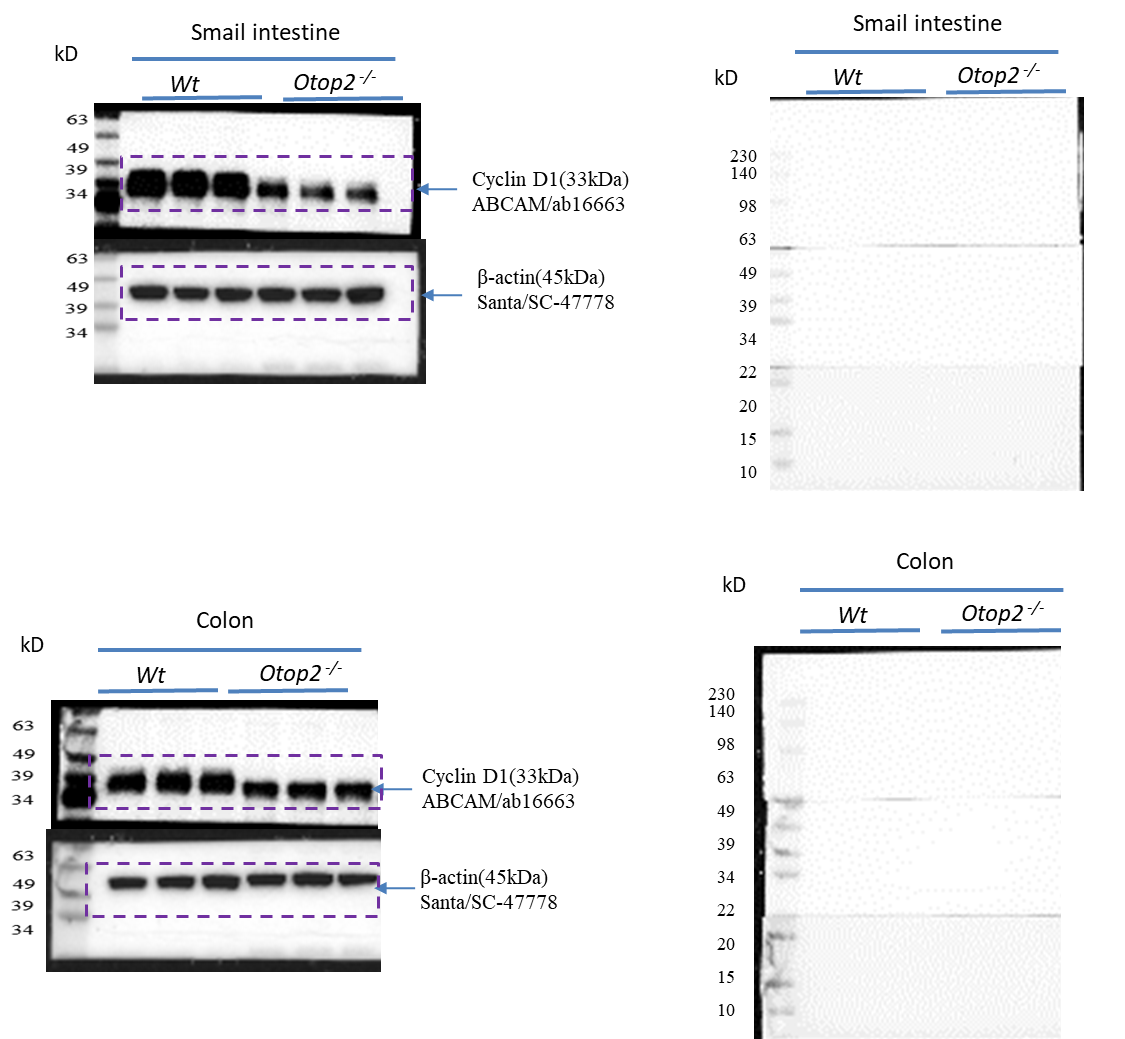
**

**Orignal Figure 10D**


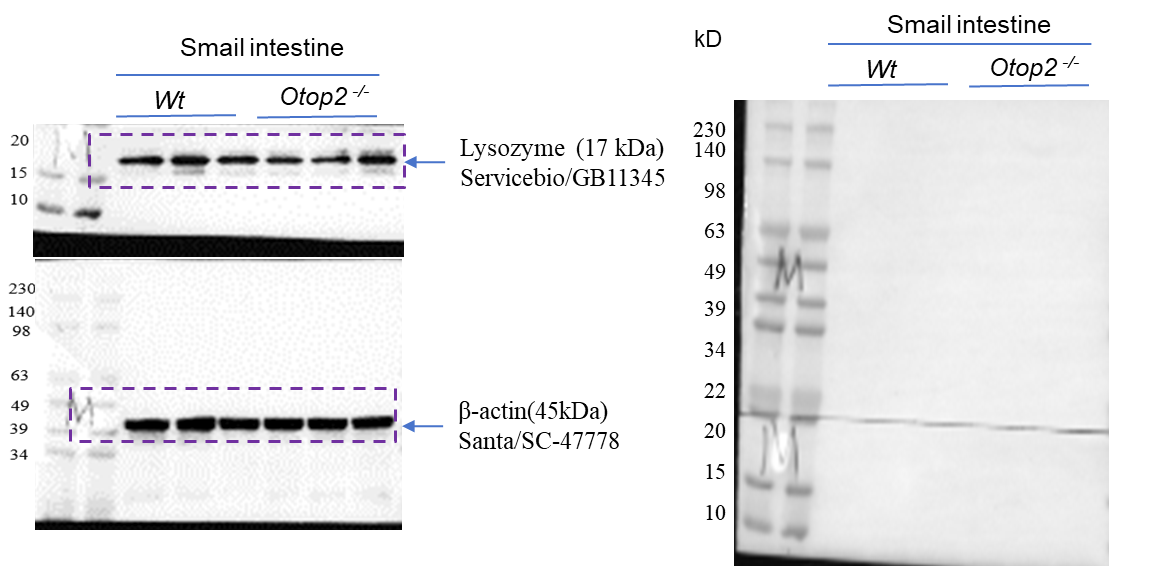


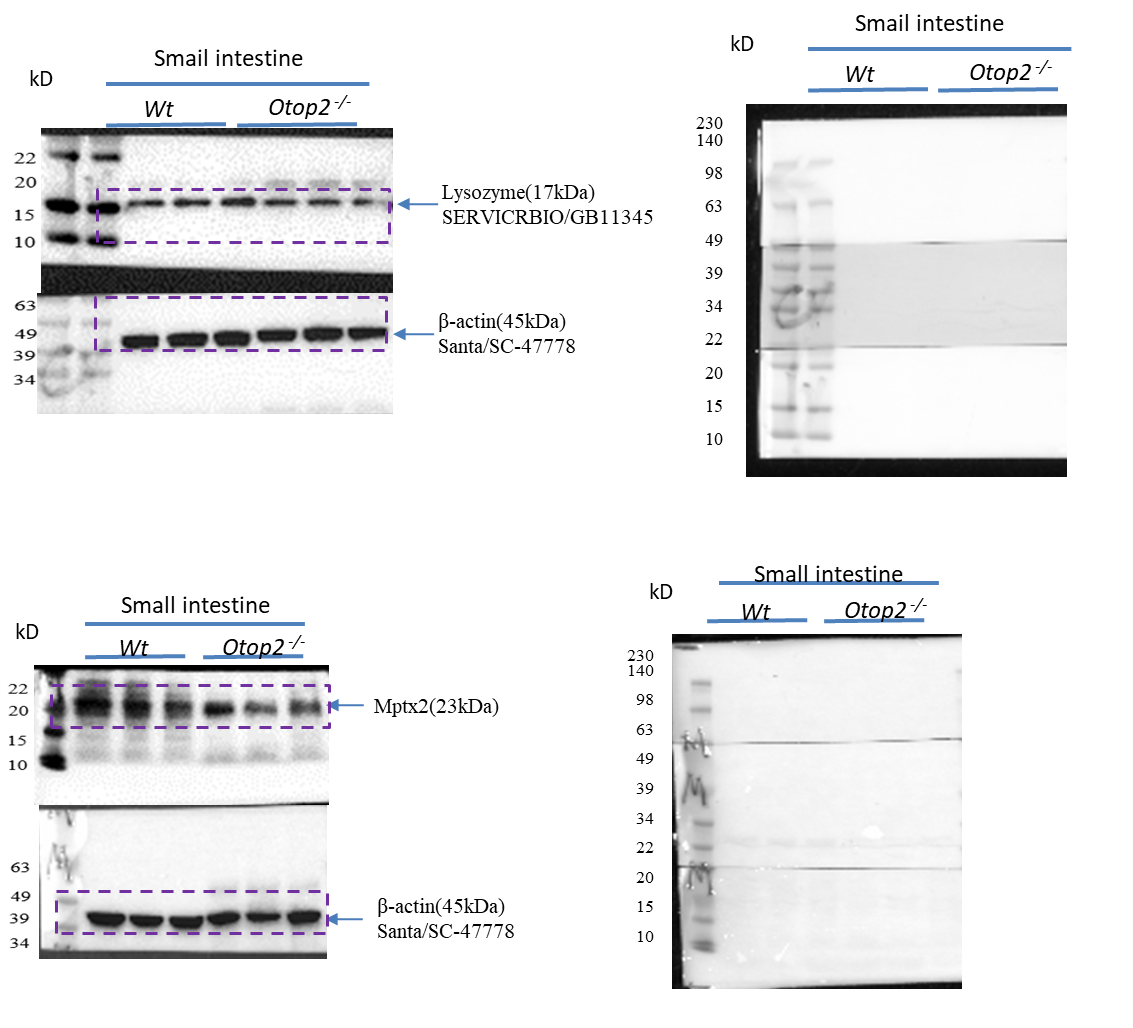


**Orignal Figure 10D**


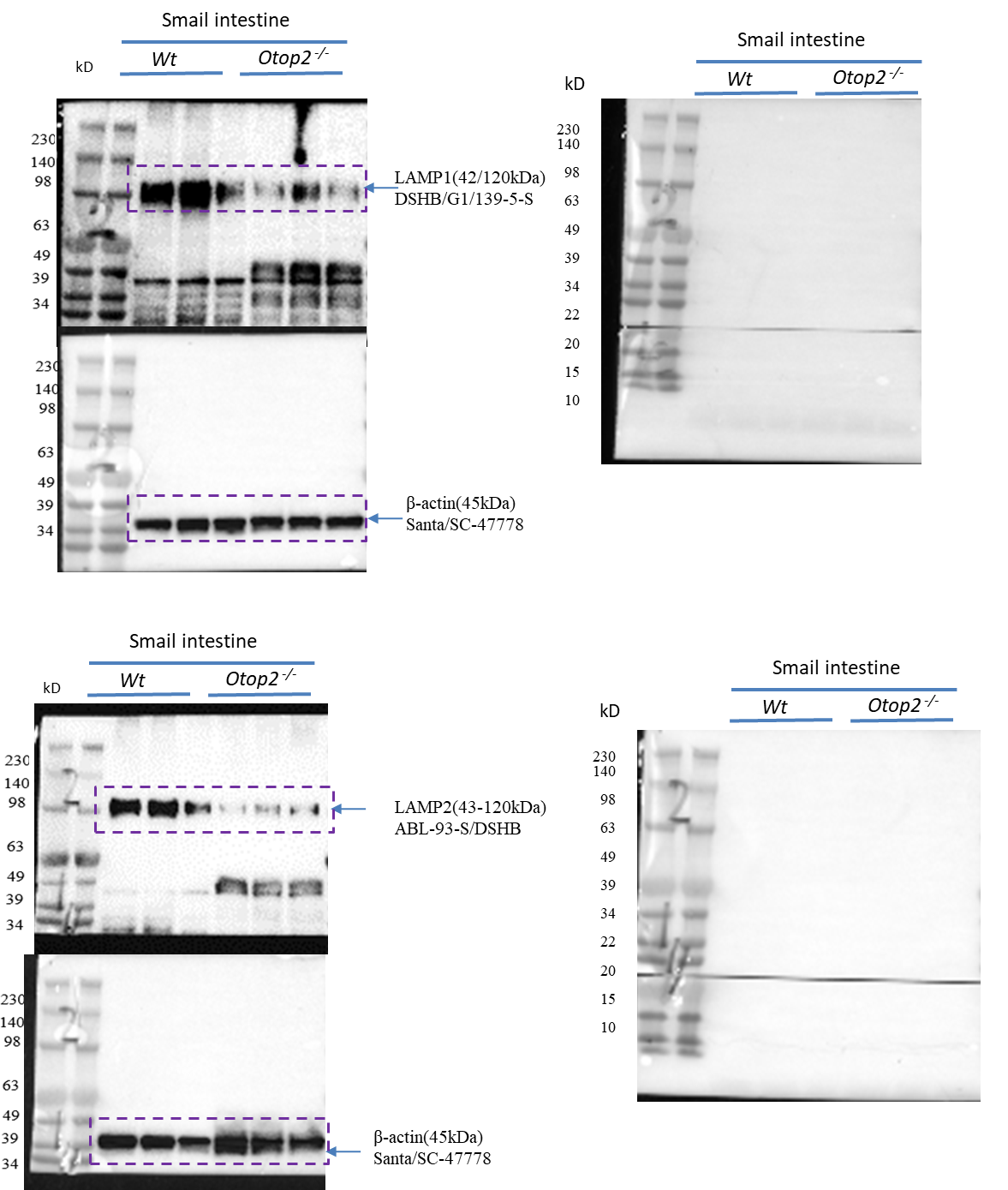


**Orignal Figure 11C**


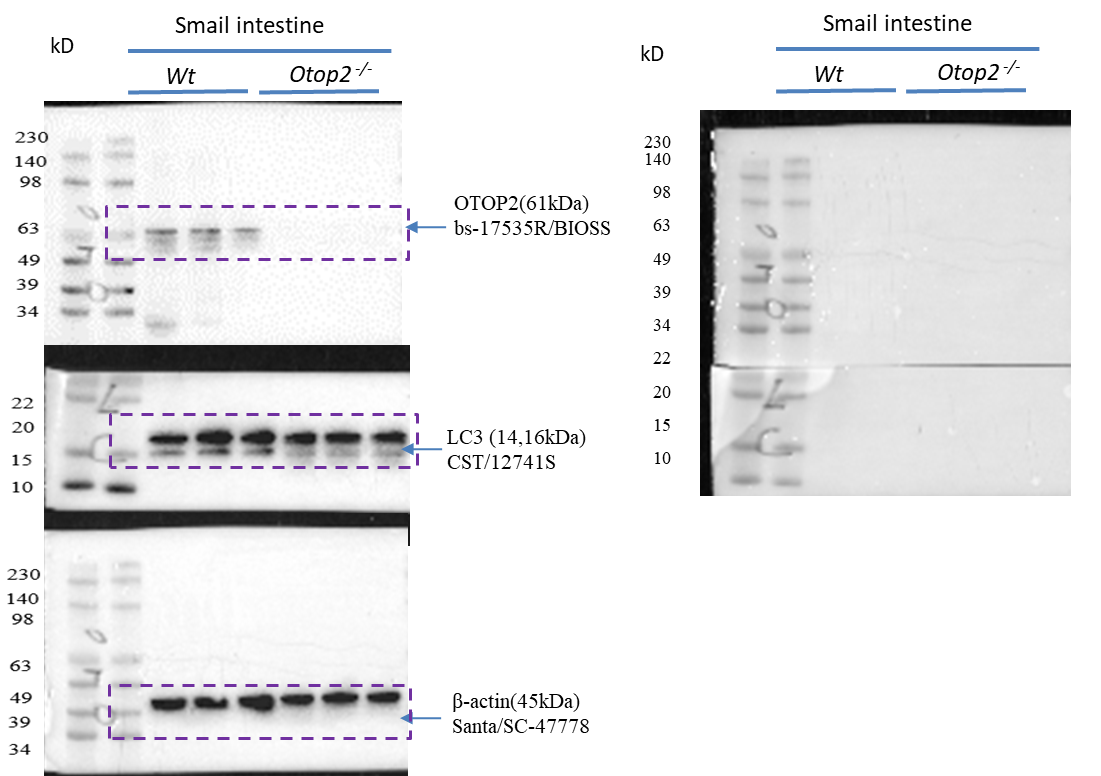


**Orignal Figure 12A**


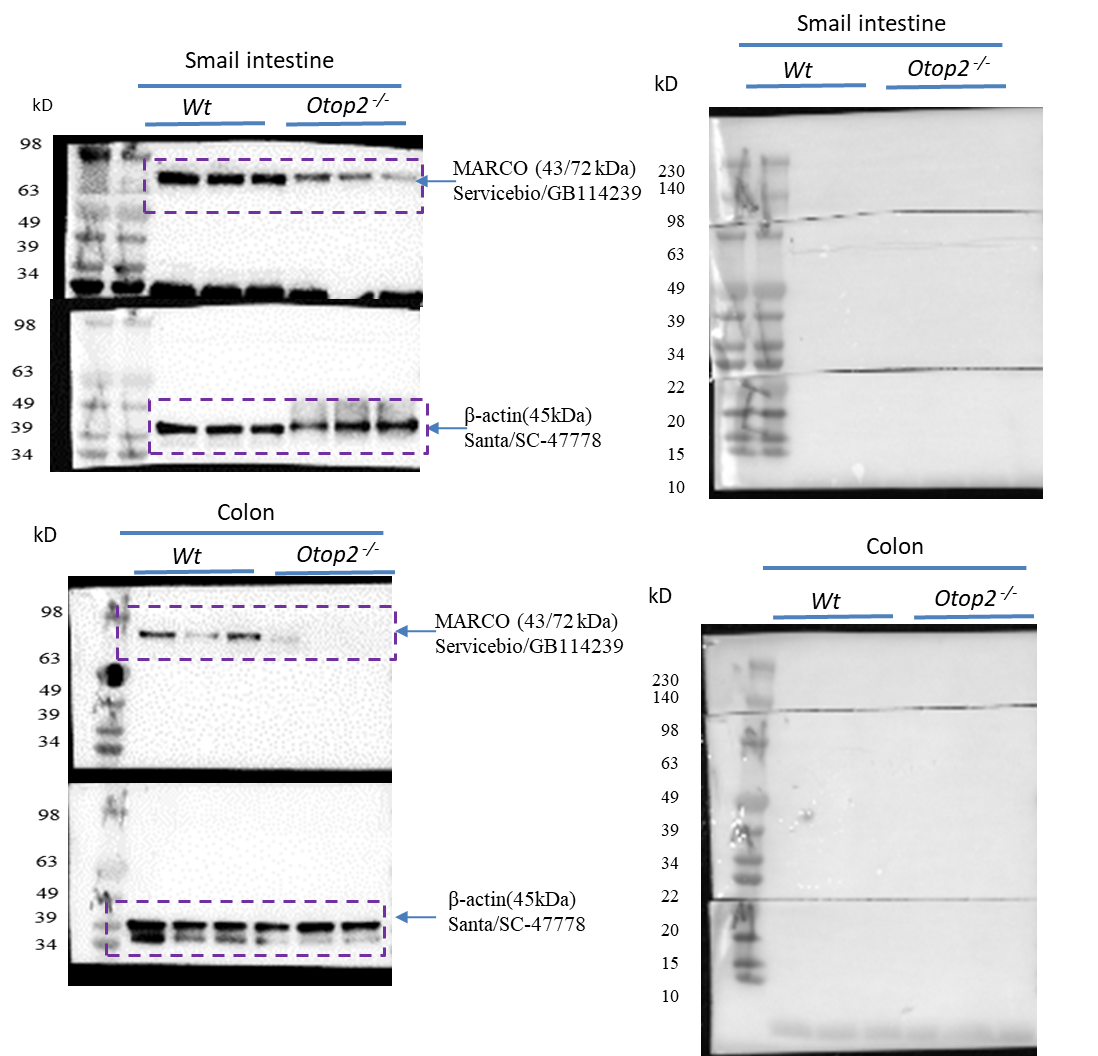


**Orignal Figure 12E**

**
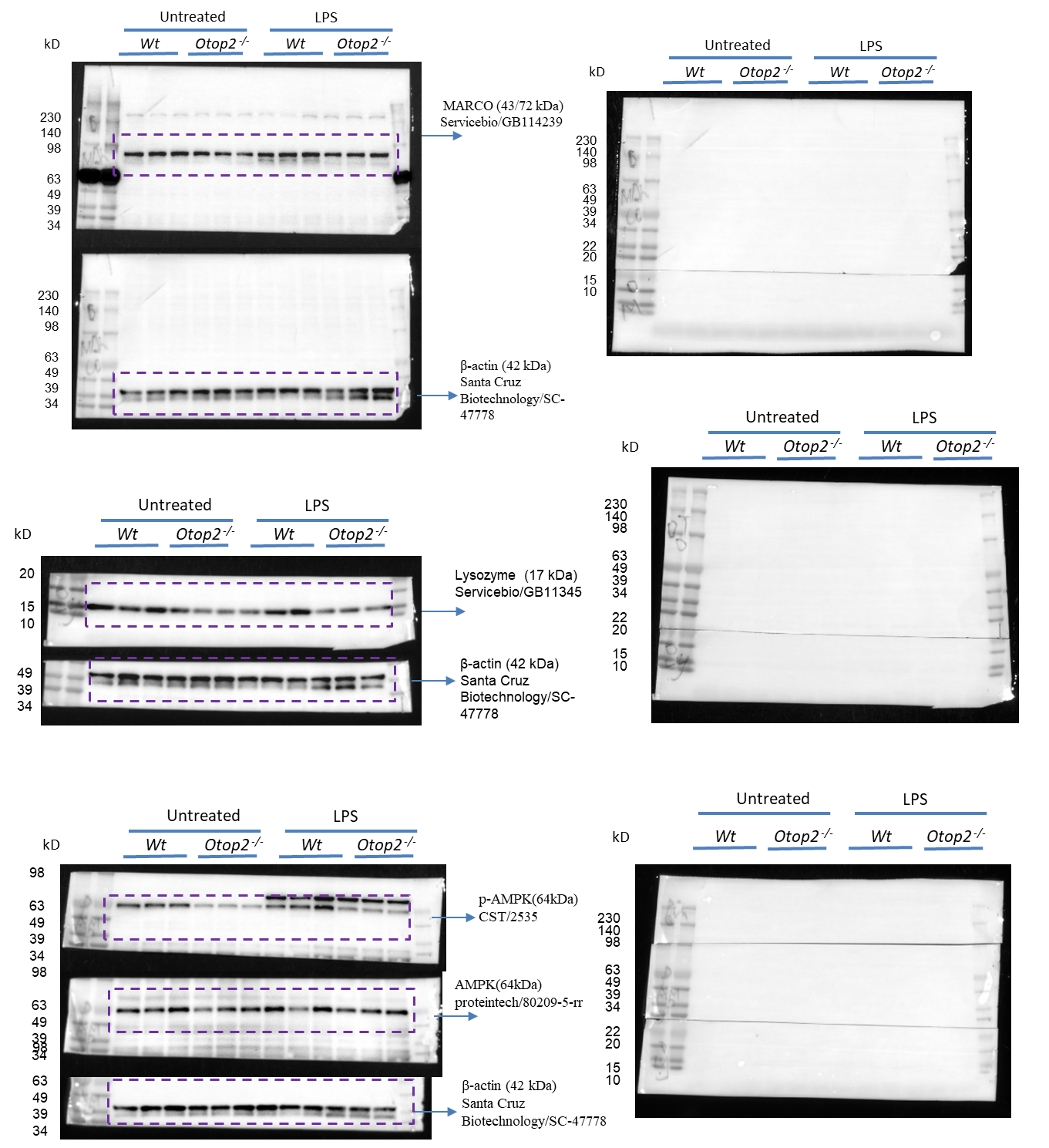
**

**Orignal Figure 12E**

**
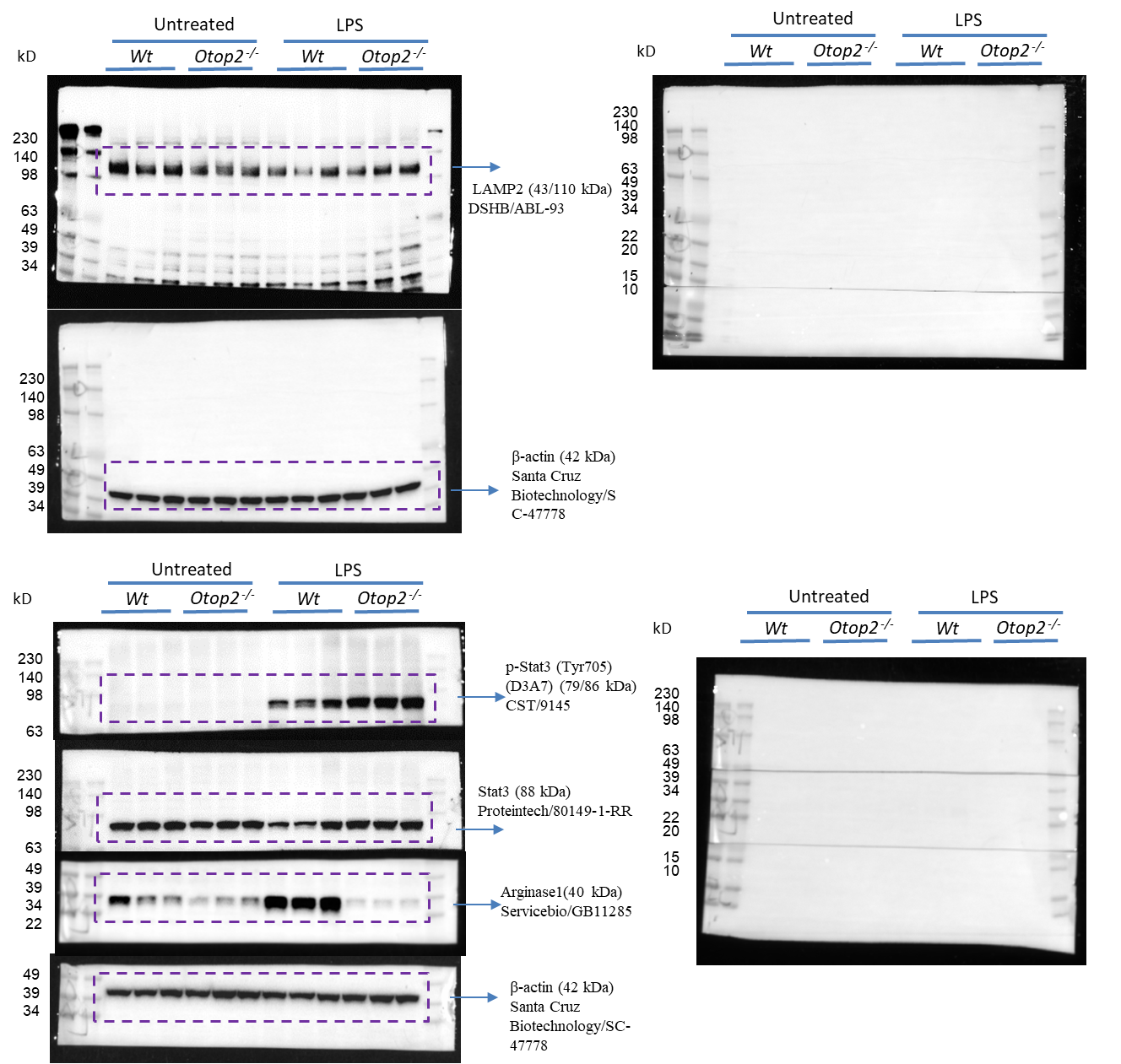
**

**Orignal Figure 13D**


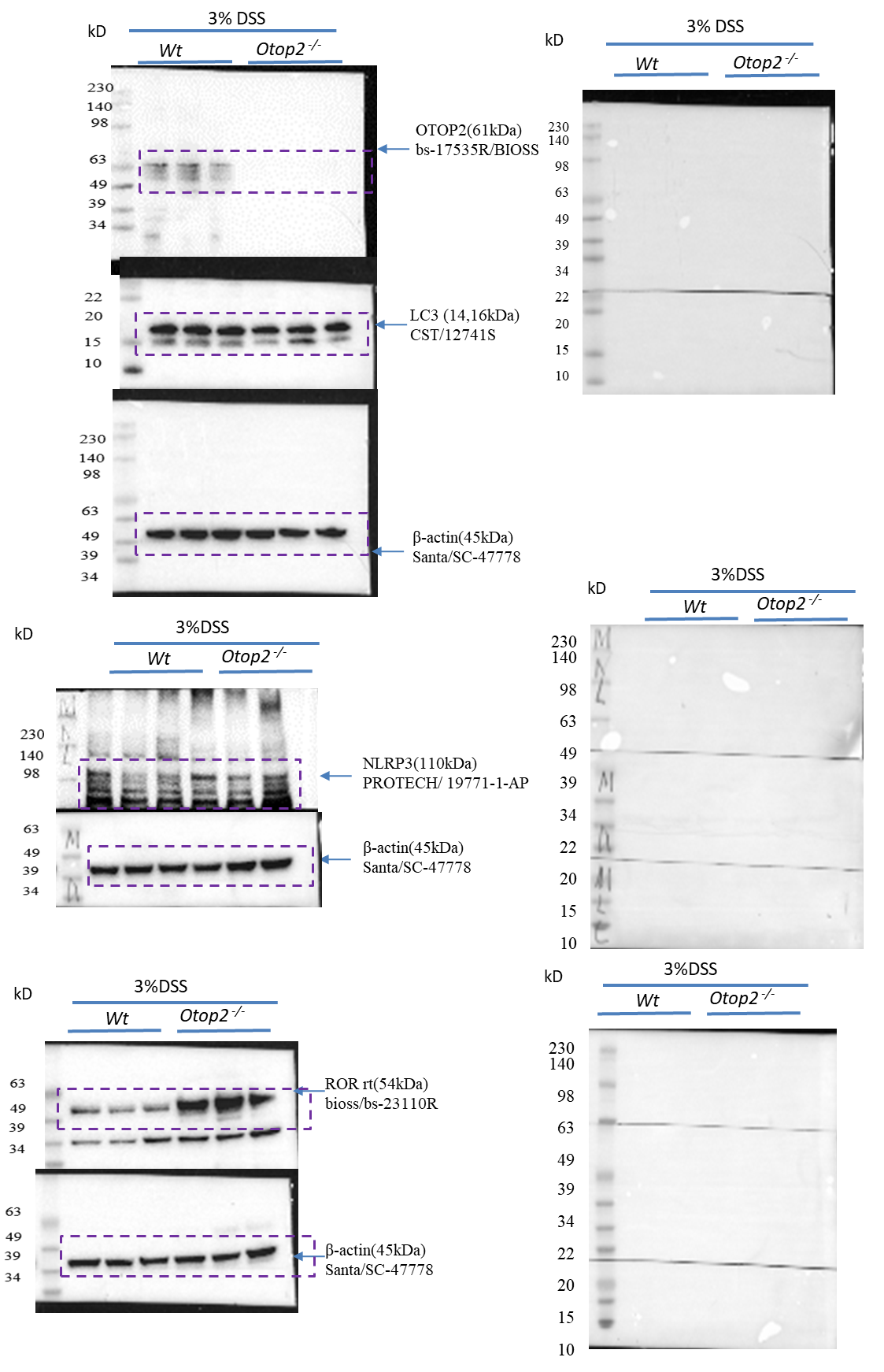


**Orignal Figure 13D**


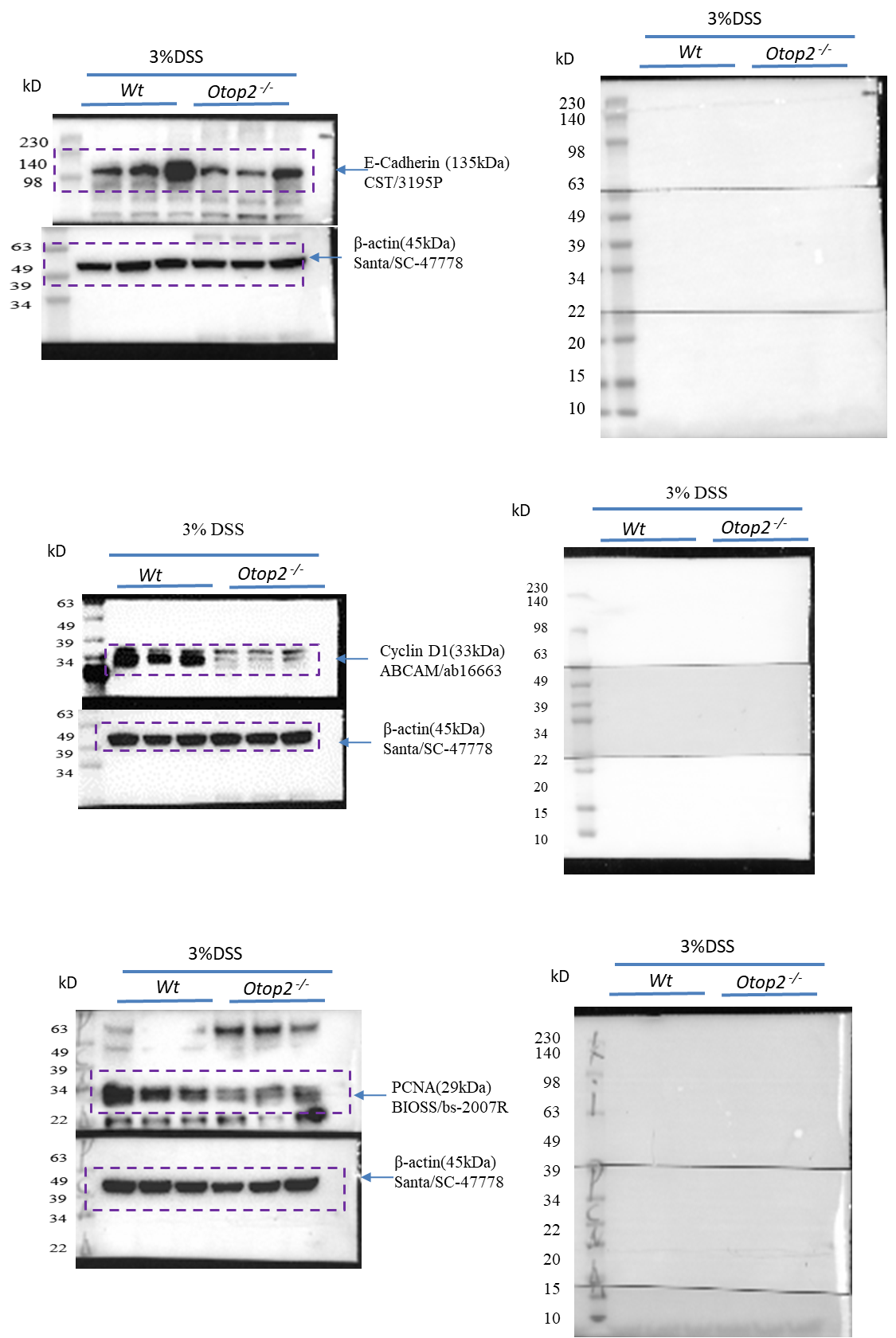


**Orignal Supplementary Figure 5C**


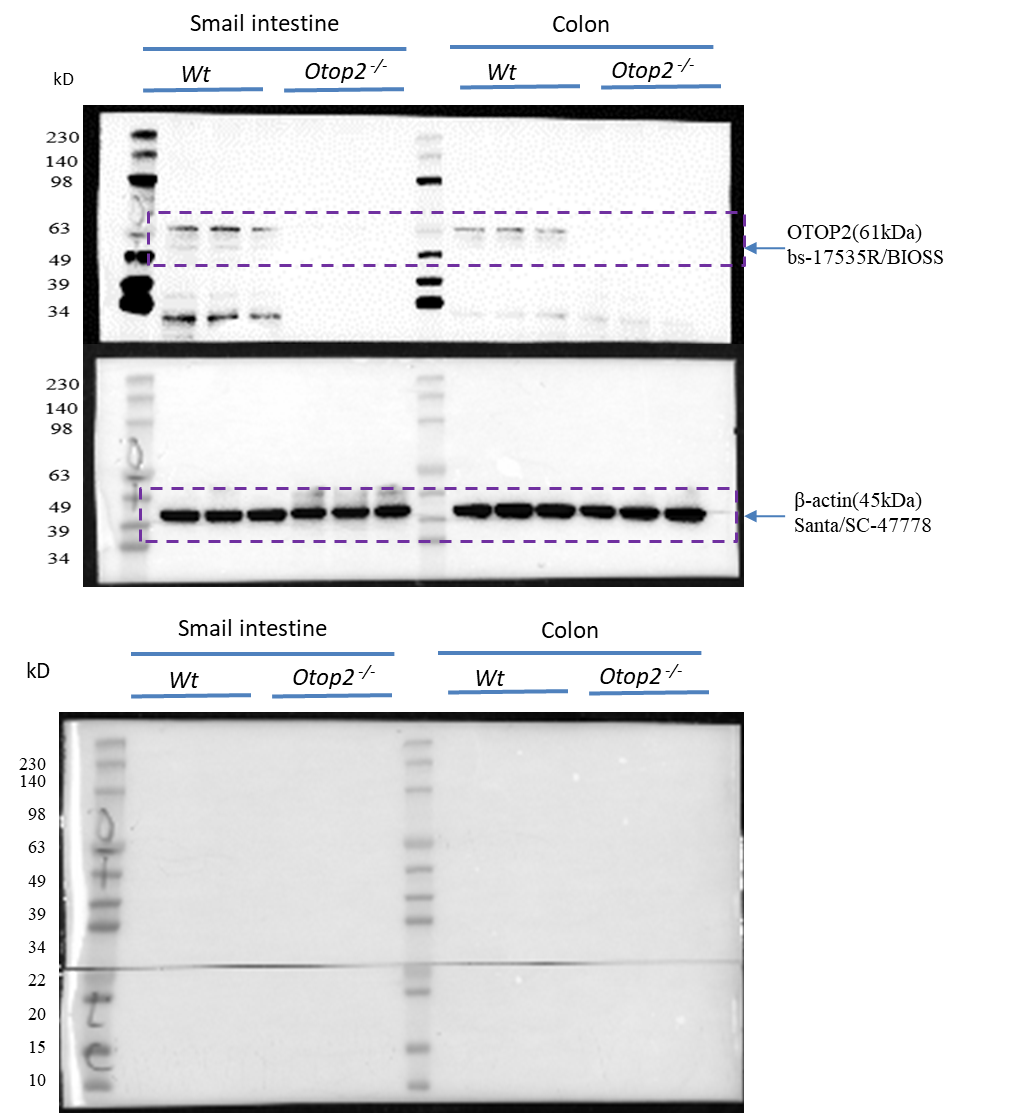

Supplement: Supplements Information [file mmc2.docx]
